# Prediction of single nucleotide polymorphisms of RNA dependent RNA polymerase for the potato leafroll virus using computational and experimental approaches

**DOI:** 10.1038/s41598-025-14436-8

**Published:** 2025-08-17

**Authors:** Dalia G. Aseel, Ayaat M. Elmaghraby, Ali El-Far

**Affiliations:** 1 Plant Protection and Biomolecular Diagnosis Department, City of Scientific Research and Technological Applications (SRTA, City), Arid Lands Cultivation Research Institute (ALCRI),, New Borg El-Arab, 21934 Egypt; 2 Nucleic Acid Research Department, Genetic Engineering and Biotechnology Research Institute, City of Scientific Research and Technological Applications (SRTA, City), Alexandria, Egypt; 3 Department of Biochemistry, Faculty of Veterinary Medicine, Damanhour University, Damanhour, 22511 Egypt

**Keywords:** PLRV, RdRp, SNPs, Protein physicochemical properties, Computational biology and bioinformatics, Plant sciences

## Abstract

Potato leafroll disease, caused by potato leafroll virus (PLRV), is one of the most devastating diseases, resulting in significant losses to the potato crop. Research on comprehensive studies examining the prediction of mutations in PLRV genes for this viral disease is limited. This study aims to quantify and characterize the accumulation of these genetic mutations by predicting SNPs for PLRV RNA-dependent RNA polymerase (RdRP) using computational approaches. We utilized advanced online protein prediction tools to explore the impact of DNA mutations on protein function and structure. These tools enabled us to assess the potential level and location of disorder within proteins, providing crucial insights into their behavior. In our study, the polymerase chain reaction (PCR) yielded a product corresponding to the PLRV-RdRp gene, measuring 450 base pairs (bp). This sequence has been documented in GenBank under the access number MT576073 and is cataloged in the UniProt database with the ID A0A8E6I3S8. The phylogenetic analysis of the PLRV-RdRp sequence involved a comparative study with reference sequences in the NCBI database. We focused on two notable predicted inversion mutations located at positions 93 (A93T) and 117 (K117G). Remarkably, the (Lys/Gly) mutation at position 117 exhibited a substantial disorder percentage of 61%, significantly higher than the reference sequence’s 13.4%. These single nucleotide polymorphisms (SNPs) are not just statistical anomalies; they have real implications. They alter RdRp protein stability and physicochemical properties, altering pocket residues and influencing the protein’s interaction with natural compounds. In conclusion, our findings provide a solid foundation for future research and development efforts focused at the rapid diagnosis and sustainable management of PLRV.

**Supplementary Information:**

The online version contains supplementary material available at 10.1038/s41598-025-14436-8.

## Introduction

**Fig. 1:**
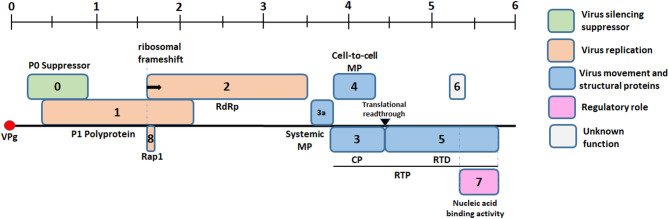
Potato leafroll virus (PLRV) genome organization with positive-sense single-stranded RNA genome. Genome and ORFs are drawn to scale.

Potato leaf rolling is a complex process influenced by various factors, including phloem blockage, osmotic stress, hormonal imbalances, and structural changes in leaf cells. These factors lead to the distinct upward rolling of potato leaf cells. This symptom indicates a physiological disturbance in the plant and can facilitate aphid feeding, thereby promoting the spread of viruses^1,2^. Among viruses is the potato leafroll virus (PLRV), which belongs to the genus *Polerovirus* and the family *Luteoviridae*. PLRV has a genome of positive-sense single-stranded RNA that measures between 5.3 and 5.9 kb. This genome comprises six to eight principal open reading frames (ORFs), which are numbered from 0 to 8^3–5^. Open reading frames code for essential proteins with functions in replication (P1, P2, P8) and gene silencing suppression (P0). The necessary component for replicating the PLRV genome is P1, a proteinase-containing polyprotein. Its self-cleavage releases VPg, a protein linked to the viral genome at both the 5′ end and the 3′ end without a poly (A) tail in OH group^6,7^. The conserved motifs characteristic of RNA-dependent RNA polymerase (RdRp) are delivered by P1, which is produced through a rare ribosomal frameshifting from ORF1 to ORF2, encoding P2^8^. ORFs 3a, 3, 4, and 5 code for proteins involved in systemic movement protein (MP) function in plants. ORF 3 encodes the coat protein (CP) while stochastic ribosomal readthrough of the CP stop codon fuses the readthrough domain (RTD), which is encoded by ORF 5, to produce the readthrough protein (RTP). The CP and RTP together form the icosahedral viral capsid, with the N-terminal portion of the RTD being the key structural determinant of aphid transmission. ORF 7 produces a protein that functions in a regulatory capacity, providing feedback regulation through binding to the RNA genome, whereas the function for the protein coded by ORF 6 is unknown. ORF 8 produce a replication-associated protein1 (Rap1)^9^ (Fig. 1).

Mutations found in the segment that encodes RdRp, CP, and VPg, along with a non-coding intergenic region situated between ORF2 and ORF3 at the 5’ UTR of subgenomic RNA 1, typically lead to the emergence of poleroviruses^7,10–13^. Grasping the molecular mechanisms of these interactions paves the way for novel strategies to manage viral infections that significantly threaten agriculture^14^. New viral strains exhibiting significant traits arise due to viral evolution, driven by RNA recombination and the beneficial buildup of mutations in specific genome regions^7,11,15,16^. Previous studies have shown that poleroviruses exhibit increased single-nucleotide polymorphisms (SNPs) in the ORFs encoding P0, P1, and CP-RTD. In contrast, SNPs are less frequent in the ORFs for P2 through P4, except for a conserved region of P2 located within the P1–P2 boundary^7,17,18^. Mutations can affect several proteins because the *polerovirus* genome contains overlapping ORFs^7^. Nonetheless, a comprehensive profile of variation in the *polerovirus* genome is lacking^7^.

Intrinsically disordered proteins (IDPs) are functional proteins that lack stable tertiary structures in physiological conditions^19^. The protein’s function and the role of IDP are influenced by its structure. Recently, many studies have emphasized the significance of unstructured or disordered regions in determining a protein’s function^20^. Disordered proteins play a crucial role in key cellular functions, including signaling, cell regulation, survival, differentiation, proliferation, and apoptosis^21,22^. Investigating proteins with lengthy disordered regions often presents challenges in expressing, purifying, and crystallizing them. Consequently, computational predictive tools have been developed to forecast the degree and position of disorder within a protein^23^.

This study explores the complex dynamics of the potato leafroll virus by analyzing SNPs and mutations in the gene coding for its RdRp. The research aims to quantify and describe the accumulation of these genetic mutations through detailed analyses. Additionally, it examines how these changes affect the RdRp’s protein composition, stability, and response to different treatment strategies. By clarifying these connections, the study aims to deepen our understanding of the virus’s behavior and its implications for control strategies.

## Materials and methods

### Virus source

**Fig. 2:**
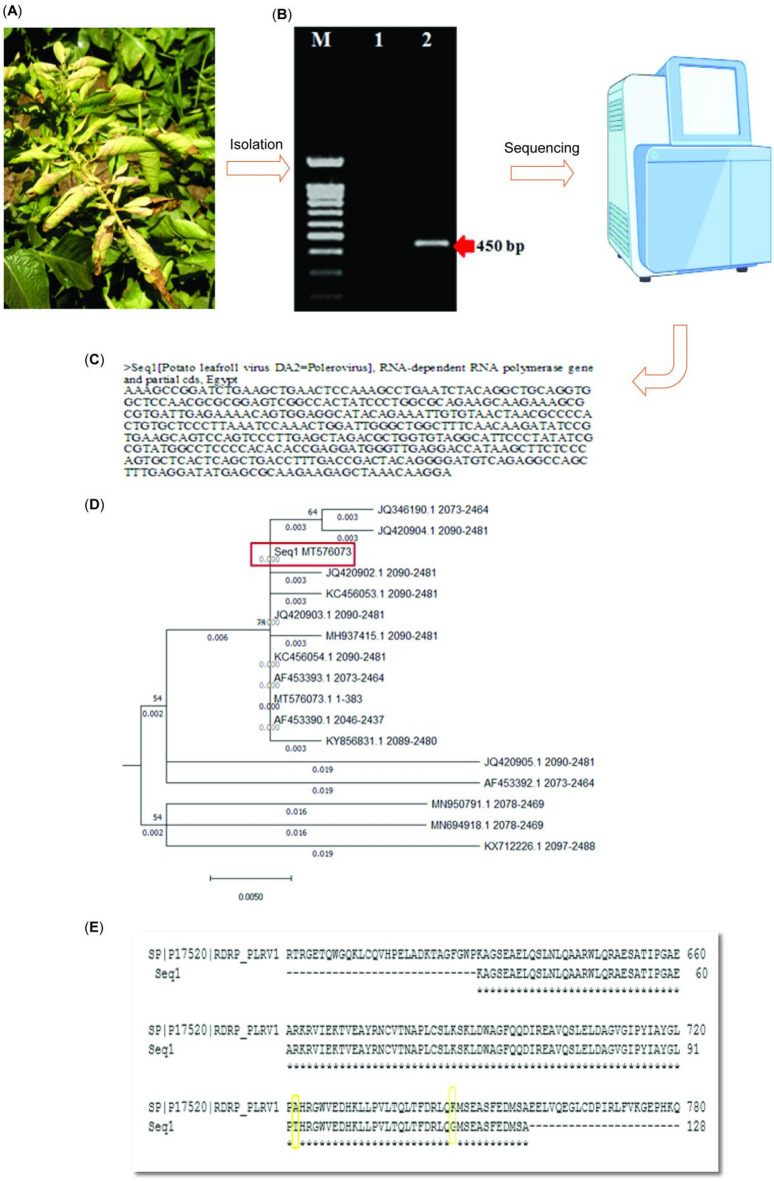
Field isolation and characterization of potato leafroll virus for RNA-Dependent RNA Polymerase (RdRp) gene. (**A**) Leaf samples show leaf roll, deformation, yellowing, and stunt symptoms. (**B**) The PCR product of the PLRV-RdRp gene is 450 bp. (**C**) Sequenced fragment of the PLRV-RdRp gene. (**D**) phylogenetic tree. (**E**) RdRp’s amino acid sequence was aligned with the reference sequence.

Leaf samples from potato plants were collected during the year of 2024 under the decision permission concerning the use and care of experimental plants at the Arid Lands Cultivation Research Institute (ALCRI) of the City of Scientific Research and Technological Applications (SRTA, City), New Borg El-Arab City, Egypt. Leaf samples showing leafroll, deformation, yellowing, and stunt symptoms (Fig. 2A) were taken from potato plants cultivated in the Borg-El Arab region, the Alexandria Governorate, Egypt. The collected leaves were washed with sterile water, dried, and stored at −20 °C for RNA extraction.

### RNA isolation and cDNA synthesis

Total RNA was extracted from 1 g of infected leaves using RNeasy Mini Kit (Qiagen, Hilden, Germany). cDNA was synthesized from the extracted RNA using a High-Capacity cDNA Reverse Transcription Kit (Applied Biosystems, Foster City, USA) according the manufacturer’s guidelines and instructions. The RT-PCR mixture was composed of RNA (3 µL, 30 ng), dNTPs (2.5 µL, 10 mM), oligo (dT) primer (5 µL, 6 pmol µL^−1^), 5X-buffer (2.5 µL), reverse transcriptase (Qiagen, Valencia, USA) (0.3 µL) and distilled water (6.7 µL). The cDNA amplification and program, as described by^[[NO_PRINTED_FORM]24^.

### Amplification of RdRp gene using specific PCR

A 1 µL of cDNA was added to 25 µL of the PCR reaction mixture (11.8 µl of Sterile Milli Q water; 2.5µL of 5 x PCR reaction green buffer; 2.5µLof 5X PCR reaction less buffer, 2.5 µL of 50mM-MgCl_2_, 2.5 µL of 10 mM-dNTPs, 1µL (10pmol/µL) of each RdRp primer sequences (forward; 5’-GTGTAACTAACGCCCCACTG-3’ and reverse; 5’-CCTTGTTTAGCTCTTCTTGCGCT-3’), 0.2 µl (5U/µL) Taq polymerase (Promega, USA). PCR was programmed with initial denaturation at 95 °C for 3 min and 34 cycles at 95 °C for 45 s, annealing at 53 °C for 45 s, and extension at 72 °C for 1 min with a final extension step at 72 °C for 5 min. PCR products were separated by electrophoresis as described by^[[NO_PRINTED_FORM]25^.

### Sequencing and phylogenetic analyses

A PCR clean-up column kit (Maxim Biotech Inc., USA) was used to purify the amplified product of the PLRV-RdRp gene according to the manufacturer’s instructions. DNA sequencing of the RdRp gene for PLRV was performed by Macrogene Company (Korea). The obtained DNA nucleotide sequence was analyzed using NCBI-BLAST (http://bast.ncbi.nlm.nih.gov/Blast.cgi) to confirm the identity of the obtained sequences. The RdRp gene sequence for PLRV was aligned with those of other *Poleroviruses* available in the GenBank database. Multiple-sequence alignments of our sequences and those published elsewhere were performed using ClustalW (1.83) according to^[[NO_PRINTED_FORM]26^. The sequences were used for comparison using MEGA 11^27^, and phylogeny was tested with the bootstrap method. The phylogenetic tree was analyzed and generated based on the UPGMA statistic method.

### Amino acid sequence prediction and alignment

The protein sequence prediction of the sequenced DNA fragment by studied isolate (seq1: accession no: MT576073). Bio-model (www.biomodel.uah.es) database was utilized as a transcription and translation tool to convert DNA sequences into RNA and subsequently into protein. The predicted amino acid sequence aligned with the reference sequence (SP|P17520|RdRp_PLRV1) (www.ensemble.org) using the multiple sequence alignment method by CLUSTAL Omega. Additionally, the sequence was aligned in the UniProt database to determine whether the detected mutations were allocated in the active site or not (www.UniProt.org).

### Characterization of the structural changes of the protein on amino acid alterations

#### Impact of recognized SNPS on amino acid alterations

PolyPhen-2^28^ and SIFT^29^ servers determined the impact of A93T and K117G mutations on amino acid alterations in the mutant protein. At the same time, Polyphen-2 predicts the variant’s effect on the protein structure and function based on sequence homology. The cut-off value for a variant to be damaging is > 0.98, and for a benign variant, it is 0.446; if the value is < 0.98 and > 0.46, the prediction will be damaging. For SIFT, the cut-off value of prediction is 0.5. A substitution with a score > 0.5 is tolerated, and < 0.5 is deleterious.

#### Impact of recognized SNPS on protein stability

I-Mutant 2.0^30^ and MUpro web servers were used to determine the impact of SNPs on protein stability. In I-Mutant 2.0, free energy change (DDG) < 0 refers to decreased stability, while DDG > 0 means increased stability. Regarding MUpro results, if ∆∆G is < 0, it means SNPs decreased protein stability; if ∆∆G is > 0, it means increased protein stability.

#### SNPs prediction disorder on PLRV-RdRp protein structure

The Phyre2.2 web server identified alterations in the 2D structure of the wild-type protein resulting from the A93T and K117G mutations^31^.

In this study, the RaptorX web server (www.raptorx.uchicago.edu) was used to identify the difference in protein structure (3D) of the predicted protein of the studied isolate and the disorder grade prediction of the two dedicated SNPs.

#### Physicochemical characteristics of wild-type and mutant proteins

Physicochemical parameters of wild-type and mutant proteins, including the number of amino acids, molecular weight, theoretical pI, amino acid composition, atomic composition, and estimated half-life, were computed using the ProtParam web server^32^.

#### Pockets’ prediction of wild-type and mutant proteins

The pockets of wild-type and mutant proteins were predicted using the DrugRep web server^33^.

#### Molecular Docking assessments for wild-type and mutant proteins

The 3D structures of wild-type and mutant proteins were generated using the I-TASSER web server^34^. Besides the generation of contact and distance maps, the generated proteins were prepared for docking using MOE 2015.10 software (ref), and the Ramachandran plot of both proteins was generated with the PROCHECK web server (https://www.ebi.ac.uk/thornton-srv/databases/pdbsum/Generate.html).

These proteins were subjected to molecular docking with the bioactive compounds of *Aloe vera*, *Artemisia campestris*, *Calotropis procera*, *Foeniculum vulgare*, *Syzygium aromaticum*, and *Thuja orientalis*, which had previously been tested against the wild-type proteins^35,36^. The 3D structures of plant active ingredients were retrieved from the LOTUS database (https://lotus.naturalproducts.net/)^37^.

Molecular docking of ligands against the wild-type protein was performed using the InstaDock v1.0 (https://hassanlab.org/instadock/) software^38^. The molecular interactions between them were visualized by BIOVIA Discovery Studio Visualizer 2016 software.

Similarly, the top 5 ligands (LTS0009009, LTS0096540, LTS0241644, LTS0123435, and LTS0087204) exhibited higher free binding energy with the wild-type and mutant proteins.

#### Similarity prediction assessment

The bioactive compound LTS0123435 exhibited the highest free binding energy with wild-type and mutant proteins. It was subjected to similarity prediction using ChemMine Tools^39^with a PubChem fingerprint search with a similarity cutoff of 0.99. The resulting compounds were then docked with wild-type and mutant proteins.

#### Molecular Docking of PLRV-RdRp’s wild-type and mutant proteins with RNAs of the initial protein and the putative movement protein of PLRV

The RNA sequences of the initial protein and the putative movement protein of PLRV were retrieved from the NCBI (https://www.ncbi.nlm.nih.gov/) from the PLRV complete genome (AY138970). The HDOCK web server determined molecular docking interactions of RdRp’s wild-type and mutant proteins with RNAs^40^.

## Results

### PCR amplification and nucleotide sequence of the PLRV-RdRp gene

The PCR product of the PLRV-RdRp gene is 450 bp (Fig. 2B) and **Supplementary File 1**. The sequenced fragment of the PLRV-RdRp gene (Fig. 2C) has been assigned an access number (MT576073) in the NCBI database.

### Phylogenetic tree analysis

PLRV-RdRp sequence isolates from Egypt compared with reference sequences from nine distinct regions which have been used for pairwise and multiple Alignments as well as phylum tree construction (KC456054.1 (China), JQ420903.1 (India), AF453393.1 (strain CU87 from Cuba), AF453390.1 (France), MH937415.1 (Germany), KY856831.1 (Argentina), KC456053.1 (China), JQ420902.1 (India), MT576073.1 (Egypt), JQ420904.1 (India), JQ346190.1 (Germany), MN950791.1 (Canada), JQ420905.1 (India), MN694918.1 (Canada), AF453392.1 (Peru), KX712226.1 (Colombia)), as shown in (**Fig. ****S1**). Unrooted phylogenetic trees based on complete sequence alignments were reconstructed. This unrooted tree illustrates relatedness of leaf roots maximum likelihood which takes a probabilistic approach to tree construction that the studied DA2 isolates (Fig. 2D). Where the most similar isolates to MT576073.1 (Egypt) were JQ346190.1 (Germany) and JQ420904.1 (India), and the little sequences in similarity were MN950791.1 (Canada), MN694918.1 (Canada) and KX712226.1 (Colombia).

### Amino acid sequence prediction and alignment

The predicted amino acid of the sequenced fragment of the PLRV- RdRp gene using **(**www.biomodel.uah.es**)** is:

**Table:** 

>RdRp_PLRV Protein P1-P2 =Potato leafroll virus=protein_id=“QVP25866” KAGSEAELQSLNLQAARWLQRAESATIPGAEARKRVIEKTVEAYRNCVTNAPLCSLKSKLDWAGFQQDIREAVQSLELDAGVGI PYIAYGLPTHRGWVEDHKLLPVLTQLTFDRLQGMSEASFEDMSA		

The predicted amino acid sequence was aligned with the reference sequence (SP|P17520|RdRp_PLRV1) (Fig. 2E) using the multiple sequence alignment method by CLUSTAL Omega, available at www.ensemble.org. The alignment shows inversion mutations in positions 93 (A93T) and 117 (K117G) in the RdRp protein. The sequence alignment in UniPort (www.uniprot.org) revealed that the location of the targeted sequence is either too far or not located within the enzyme’s active sites, which is indicated in red. It is too far with the amplified sequence.

The data illustrated in Fig. S2 represented the contact and distance maps, as well as the Ramachandran plots of generated 3D structures of wild and mutant proteins, with the most favored regions at 75.4% and 74.3%, respectively.

### Effect of SNPs on rdrp’s amino acids alterations

The data in Table 1 illustrate the impact of recognized SNPs on amino acid changes. PolyPhen-2 indicated that A93T and K117G had a benign effect on amino acids, while predictions from the SIFT web server showed deleterious effects of these SNPs.

**Table 1:** Functional impact analysis of genetic variants via bioinformatics tools.

Amino acid changes	Impact of amino acid alteration		Impact on protein stability		
	PolyPhen-2	SIFT	I-Mutant2.0		MUpro
	Impact (score)	Impact	Stability	DDG	Stability
A93T	Benign (0.001)	Deleterious	Decrease stability	−0.96	Decrease stability
K117G	Benign (0.004)	Deleterious	Decrease stability	−0.72	Decrease stability

Polyphen-2 predicts the variant’s impact on the protein structure and function based on sequence homology. The cut-off value for a variant to be probably damaging is > 0.98, and for a benign variant, it is 0.446; if the value is < 0.98 and > 0.46, the prediction is possibly damaging.

For SIFT, the cut-off value of prediction is 0.5. A substitution with a score > 0.5 is tolerated, and < 0.5 is deleterious.

For I-Mutant2.0, free energy change (DDG) < 0 decreases stability, while DDG > 0 increases stability.

MUpro, if ∆∆G < 0, means decreased stability; if ∆∆G > 0, it means increased protein stability.

### Effect of SNPs on rdrp’s protein stability

Using I-Mutant 2.0 and MUpro web servers, the prediction of the effect of A93T and K117G SNPs on RdRp’s protein stability revealed that these SNPs decreased protein stability, as shown in Table 1.

### Effect of SNPs on the 2D structure of rdrp’s protein

**Fig. 3:**
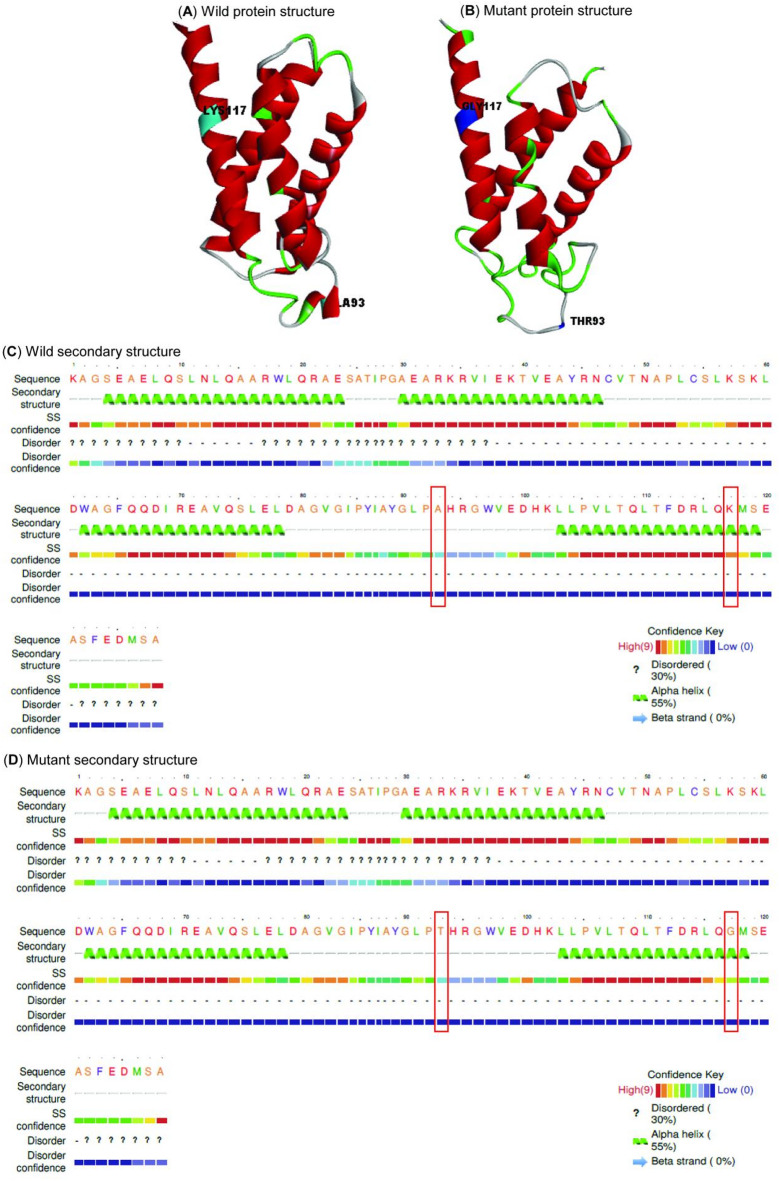
Three-dimensional (3D) and 2D structures of wild-type and mutant proteins (**A**) 3D structures of wild protein. (**B**) 3D structures of mutant protein. (**C**) 2D structures of wild protein. D) 2D structures of mutant protein.

The data illustrated in Fig. 3a and b show the 3D structures of wild and mutant proteins generated by the I-TASSER web server, which are used for pocket prediction and molecular docking assessments.

A93T and K117G SNPs caused changes in the 2D structure of the RdRp protein, resulting in the S amino acid residue at position 119 no longer participating in the 4th *α*-helix of the protein structure (**Fig. 3C**and **3D**).

### Effect of SNPs on rdrp’s protein disorder

**Fig. 4:**
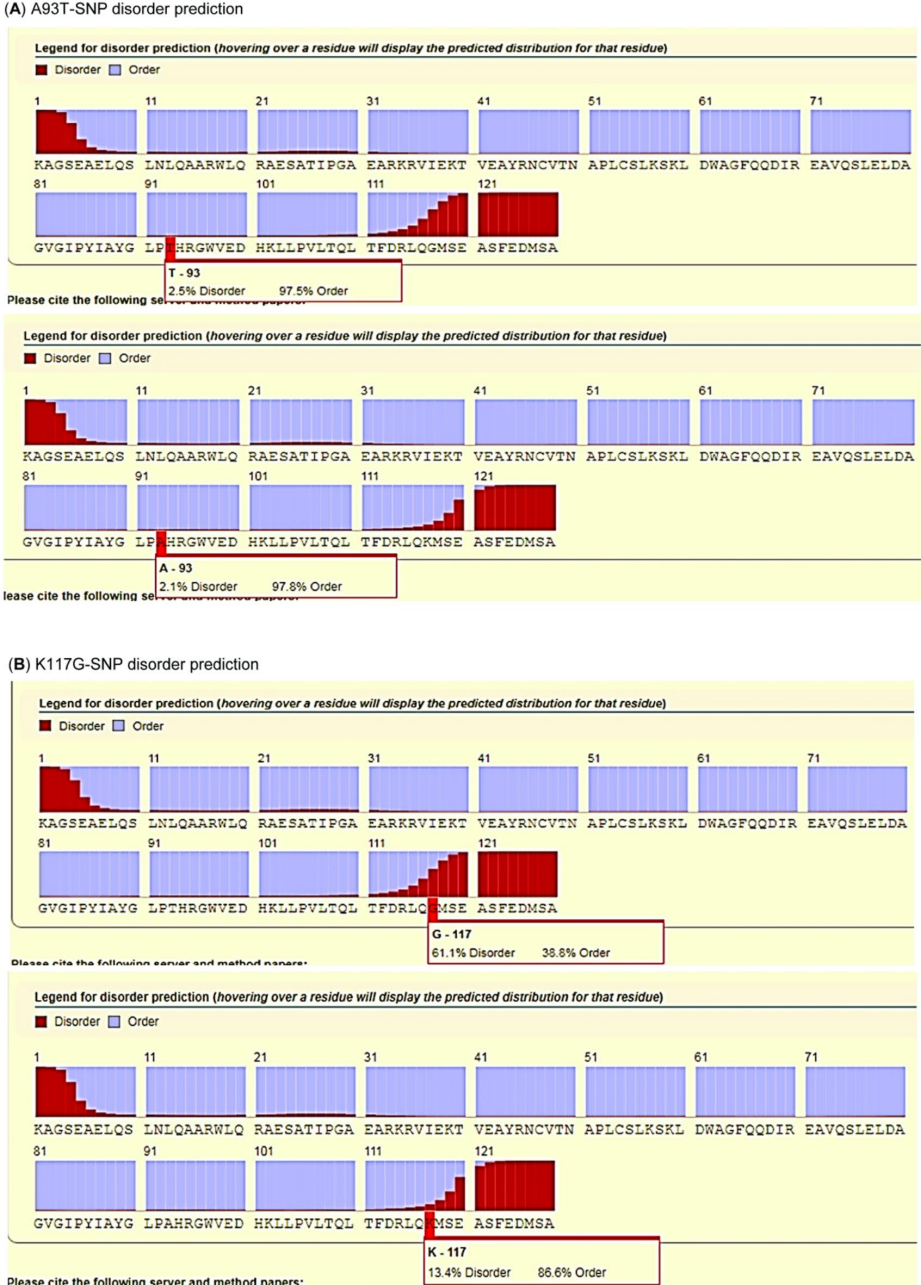
Effect of SNPs on PLRV-RdRp’s protein disorder. (A) A93T-SNP disorder prediction. (B) K117G-SNP disorder prediction.

In this study, www.raptorx.uchicago.edu was used to identify the difference in protein structure (3D) of the predicted protein of the studied sequence isolates and the reference protein sequence structure (Fig. 4A and B). The disorder grade prediction of the two dedicated SNPs on the studied isolate and the reference sequence with the wild-type amino acid chain illustrates that the isolate exhibits the highest disorder in protein function compared to the reference wild-type. As the studied sequences have 2.5% disorder, whereas the wild-type sequence reference has 2.1% disorder in SNP (A93T), the low probability of disorder may stem from the fact that they have similar 3D shapes. On the other hand, the studied isolate mutation disorder had the highest disorder in the function of the protein than the reference wild-type, as the studied sequence had 61% disorder, while in the wild-type sequence reference is 13.4% disorder in SNP (K117G) indicating that this SNP with high disorder effect because of lysine have electrically charged side chain. Still, the glycine has a no-polar side chain.

### Effect of SNPs on rdrp’s protein physicochemical properties

Analyzing the 3D structures of wild-type and mutant proteins using the ProtParam web server revealed that these SNPs altered the physicochemical characteristics of the wild-type, as shown in Table 2. The molecular weight of the wild-type is 14215.17, while that of the mutant protein is 14174.07 Da. The theoretical pIs were also 5.71 and 5.39 for the wild-type and mutant proteins, respectively. Furthermore, the percentages of A (from 17.13.3–16.12.5%), T (from 5.3.9–6.4.7%), G (from 7.5.5–8.6.2%), and K (from 7.5.5–6.4.7%) in the amino acid composition were modified due to these SNPs. Concurrently, the total number of positively charged residues decreased from 15 to 14 amino acid residues, and with atomic composition, structural formula, total number of atoms, aliphatic index, and grand average of hydropathicity (GRAVY) were changed.

**Table 2:** ProtParam computed physicochemical parameters of wild-type and mutant proteins.

Parameters			Wild-type protein	Mutant protein
Number of amino acids			128	128
Molecular weight (Da)			14215.17	14174.07
Theoretical pI			5.71	5.39
Amino acid composition (number, percentage)		Ala (A)	17, 13.3%	16, 12.5%
		Arg (R)	8, 6.2%	8, 6.2%
		Asn (N)	3, 2.3%	3, 2.3%
		Asp (D)	6, 4.7%	6, 4.7%
		Cys (C)	2, 1.6%	2, 1.6%
		Gln (Q)	8, 6.2%	8, 6.2%
		Glu (E)	11, 8.6%	11, 8.6%
		Gly (G)	7, 5.5%	8, 6.2%
		His (H)	2, 1.6%	2, 1.6%
		Ile (I)	5, 3.9%	5, 3.9%
		Leu (L)	15, 11.7%	15, 11.7%
		Lys (K)	7, 5.5%	6, 4.7%
		Met (M)	2, 1.6%	2, 1.6%
		Phe (F)	3, 2.3%	3, 2.3%
		Pro (P)	5, 3.9%	5, 3.9%
		Ser (S)	9, 7.0%	9, 7.0%
		Thr (T)	5, 3.9%	6, 4.7%
		Trp (W)	3, 2.3%	3, 2.3%
		Tyr (Y)	3, 2.3%	3, 2.3%
		Val (V)	7, 5.5%	7, 5.5%
		Pyl (O)	0, 0.0%	0, 0.0%
		Sec (U)	0, 0.0%	0, 0.0%
		Total number of negatively charged residues (Asp + Glu)	17	17
		Total number of positively charged residues (Arg + Lys)	15	14
Atomic composition		Carbon (C)	628	625
		Hydrogen (H)	1001	994
		Nitrogen (N)	177	176
		Oxygen (O)	191	192
		Sulfur (S)	4	4
Formula			C_628_H_1001_N_177_O_191_S_4_	C_625_H_994_N_176_O_192_S_4_
Total number of atoms			2001	1991
Estimated half-life	Mammalian reticulocytes (in vitro)		1.3 h	1.3 h
	Yeast (in vivo)		3 min	3 min
	*Escherichia coli* (in vivo)		3 min utes	3 min utes
Aliphatic index			90.08	89.30
Grand average of hydropathicity (GRAVY)			−0.305	−0.298

### Effect of SNPs on rdrp’s protein pockets

**Fig. 5:**
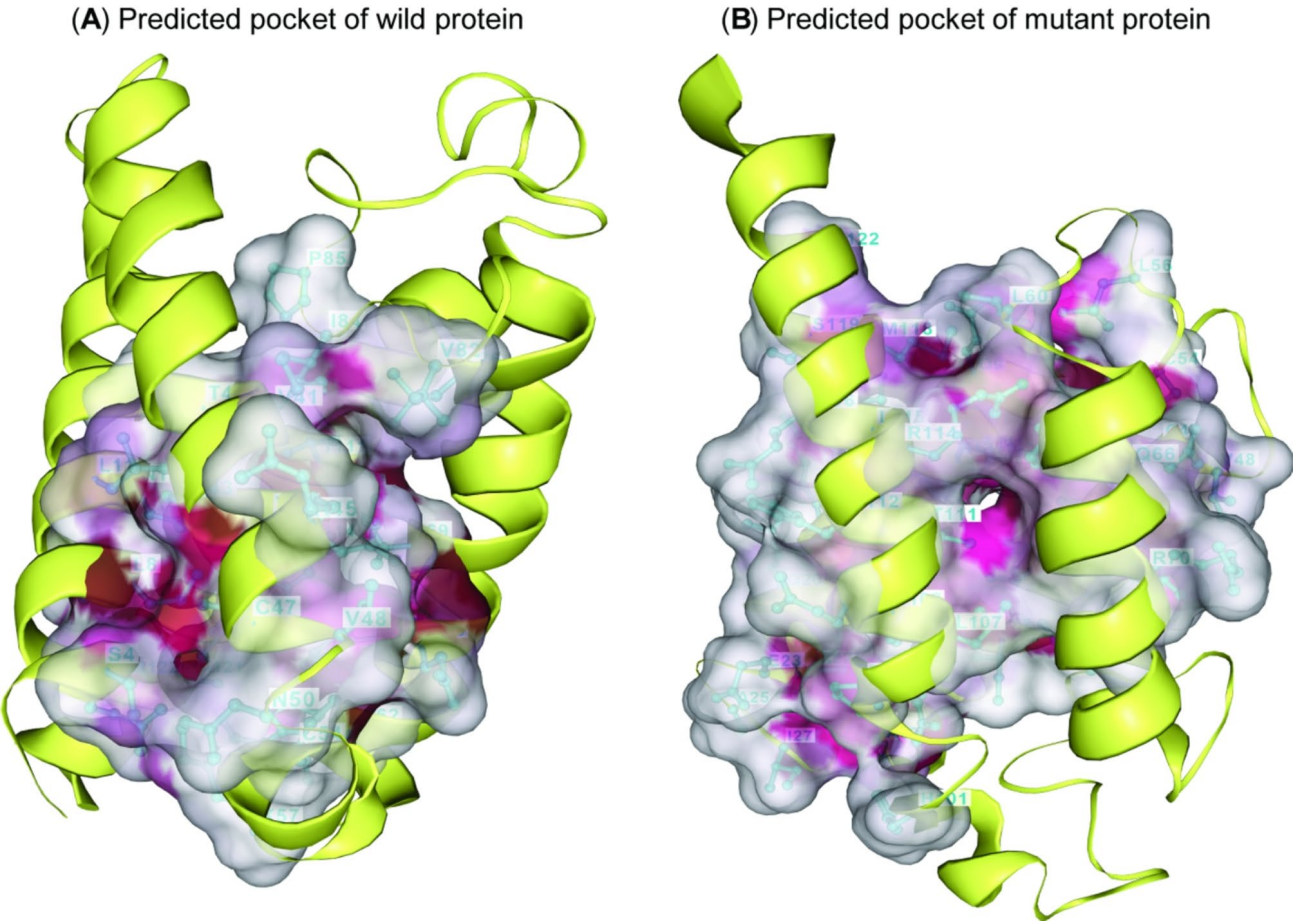
Effect of SNPs on PLRV-RdRp’s protein pockets. (**A**) The predicted pocket of the wild-type protein. (**B**) The predicted pocket of the mutant protein.

As stated in Table 3, the pockets of wild-type and mutant proteins were predicted using the DrugRep web server. SNPs altered the amino acid composition, volumes, centers, and sizes of the pockets. Predicted pocket 1 of the wild-type protein is composed of ARG45, LEU76, LEU11, ARG114, THR111, GLN66, ILE84, ALA15, ILE69, TRP62, CYS47, LEU115, GLN9, THR40, ASP61, SER4, VAL41, VAL48, SER122, VAL82, ASN12, LYS57, ASN50, LEU8, PHE65, CYS54, ALA43, GLU5, ALA121, MET118, TYR44, and PRO85 residues (Fig. 5A). In contrast, pocket 1 of the mutant is composed of GLN20, LEU19, HSD101, SER119, LEU11, GLU23, LEU13, ARG114, GLN116, ALA32, THR111, GLN66, ILE84, ALA15, ILE69, ILE27, CYS47, LEU115, ALA16, GLN9, THR40, ARG70, VAL73, PRO105, ARG33, ARG17, SER122, LEU60, VAL48, PHE112, ALA25, ALA22, ASN12, THR108, LEU8, GLN109, PHE65, CYS54, ALA43, LEU107, ILE37, MET118, LEU56, and TYR44 residues (Fig. 5B).

**Table 3:** DrugRep computed pockets of wild-type and mutant proteins.

Proteins	Pockets	Volume	Center	Size	Residues
wild-type	Pocket 1	150	44.9,50.6,59.0	9,16,12	ARG45, LEU76, LEU11, ARG114, THR111, GLN66, ILE84, ALA15, ILE69, TRP62, CYS47, LEU115, GLN9, THR40, ASP61, SER4, VAL41, VAL48, SER122, VAL82, ASN12, LYS57, ASN50, LEU8, PHE65, CYS54, ALA43, GLU5, ALA121, MET118, TYR44, PRO85
	Pocket 2	143	53.5,51.1,67.1	14,14,10	TRP18, LYS39, VAL36, GLU7, GLN14, LEU11, ARG17, SER10
	Pocket 3	72	45.4,62.7,55.6	9,12,10	PHE123, LEU115, ALA16, PHE112, LEU13, GLN116, GLN9, ARG17, ASN12, SER119
	Pocket 4	53	47.1,40.5,56.1	9,11,8	LYS39, SER4, CYS47, ALA43, GLU7, ASN50, ASN46, LEU8
	Pocket 5	150	44.9,50.6,59.0	9,16,12	TRP18, THR26, GLY29, ALA32, ARG33, ARG21
Mutant	Pocket 1	3268	54.7,55.1,57.7	19,25,23	GLN20, LEU19, HSD101, SER119, LEU11, GLU23, LEU13, ARG114, GLN116, ALA32, THR111, GLN66, ILE84, ALA15, ILE69, ILE27, CYS47, LEU115, ALA16, GLN9, THR40, ARG70, VAL73, PRO105, ARG33, ARG17, SER122, LEU60, VAL48, PHE112, ALA25, ALA22, ASN12, THR108, LEU8, GLN109, PHE65, CYS54, ALA43, LEU107, ILE37, MET118, LEU56, TYR44
	Pocket 2	180	43.0,51.1,60.6	11,15,13	TRP18, ALA30, ARG33, ILE27, VAL36, GLY29, ALA32, ALA22, THR26, ARG35, ARG21
	Pocket 3	59	45.6,43.7,55.1	10,11,9	HSD101, GLU31, TYR89, ALA32, GLU99, ALA88, ARG33, ASP100
	Pocket 4	46	49.0,56.5,45.7	10,9,9	VAL41, GLU38, ARG45, VAL82, PRO85, GLY83, GLU42, TYR86
	Pocket 5	38	61.7,45.4,54.6	9,11,7	LYS102, LEU110, LEU76, ALA72, LEU103, TRP97, SER75, VAL106

### Molecular Docking and top 5 bioactive compounds for wild-type and mutant proteins

**Fig. 6:**
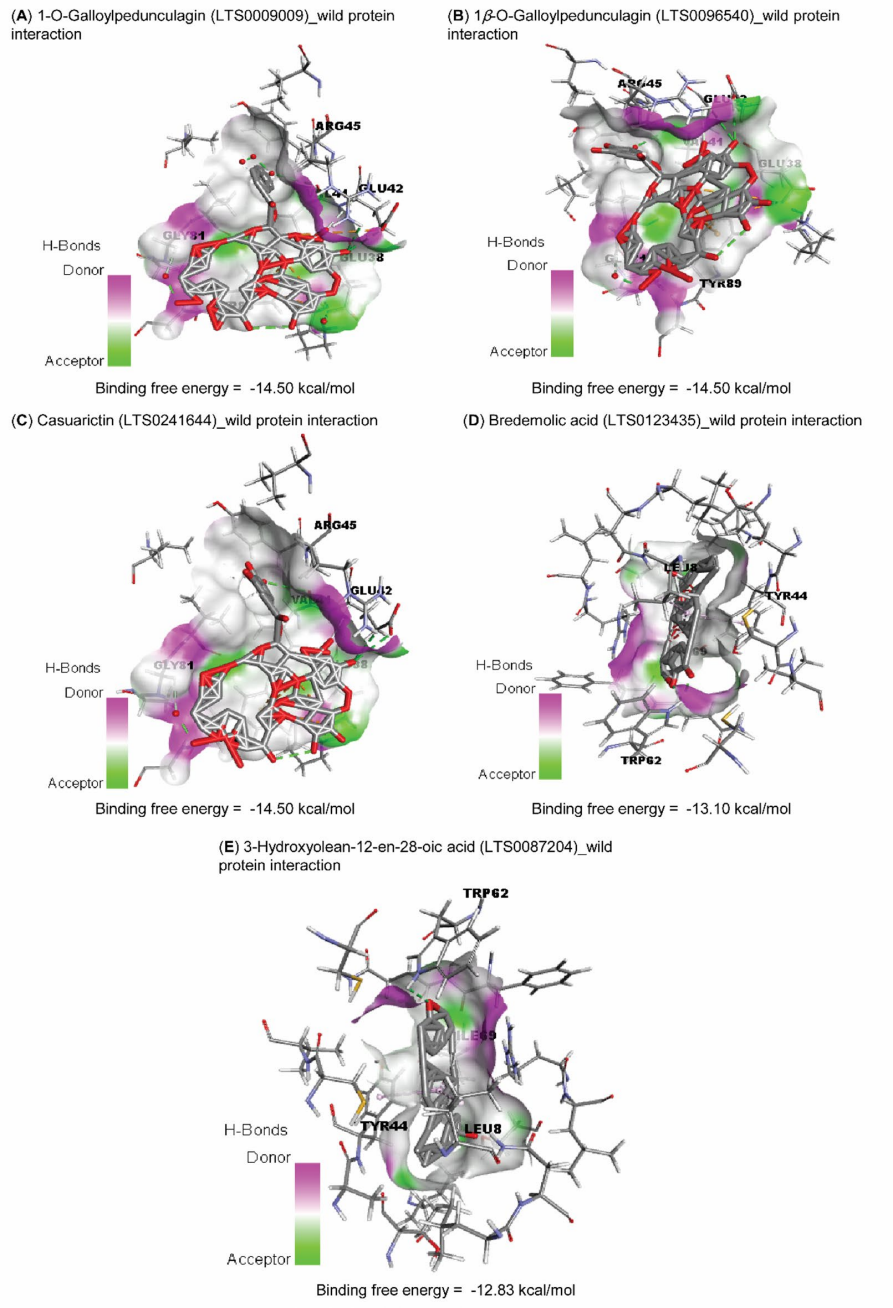
Molecular docking interactions of the top 5 bioactive compounds against the wild-type protein. (**A**) 1-O-Galloylpedunculagin (LTS0009009)_wild protein interaction. (**B**) 1*β*-O-Galloylpedunculagin (LTS0096540)_wild protein interaction. (**C**) Casuarictin (LTS0241644)_wild protein interaction. (**D**) Bredemolic acid (LTS0123435)_wild protein interaction. (**E**) 3-Hydroxyolean-12-en-28-oic acid (LTS0087204)_wild protein interaction.

The free binding energies and binding affinities (pKi) of the bioactive compound of *Aloe vera*, *Artemisia campestris*, *Calotropis procera*, *Foeniculum vulgare*, *Syzygium aromaticum*, and *Thuja orientalis* against the wild and mutant proteins are tabulated in Supplementary Table 1. Bredemolic acid (LTS0123435), 1*β*-O-Galloylpedunculagin (LTS0096540), casuarictin (LTS0241644), 1-O-Galloylpedunculagin (LTS0009009), and 3-hydroxyolean-12-en-28-oic acid (LTS0087204) exhibited the top 5 of binding free with wild type (Table 4; Fig. 6).

**Table 4:** Molecular Docking of the top 5 with wild-type and mutant proteins.

Targets	Ligand names	Ligand LOTUS IDs	Binding Free Energy (kcal/mol) ^¥^	pKi ^¥^	Interacting residues in the protein binding site
wild-type	1-O-Galloylpedunculagin	LTS0009009	−14.50	10.64	GLU38, VAL41, GLU42, ARG45, GLY81, and TYR89
	1*β*-O-Galloylpedunculagin	LTS0096540	−14.50	10.64	GLU38, VAL41, GLU42, ARG45, GLY81, and TYR89
	Casuarictin	LTS0241644	−14.50	10.64	GLU38, VAL41, GLU42, ARG45, GLY81, and TYR89
	Bredemolic acid	LTS0123435	−13.10	9.61	LEU8, TYR44, TRP62, and ILE69
	3-Hydroxyolean-12-en-28-oic acid	LTS0087204	−12.83	9.41	LEU8, TYR44, TRP62, and ILE69
Mutant	1-O-Galloylpedunculagin	LTS0009009	−13.43	9.85	ASP61, ASP68, ASP113, ARG114, and GLU120
	1*β*-O-Galloylpedunculagin	LTS0096540	−13.43	9.85	ASP61, ASP68, ASP113, ARG114, and GLU120
	Casuarictin	LTS0241644	−13.47	9.88	ASP61, ASP68, ASP113, ARG114, and GLU120
	3-Hydroxyolean-12-en-28-oic acid	LTS0087204	−11.80	8.65	GLN109 and PHE112
	Bredemolic acid	LTS0123435	−11.70	8.58	SER24, GLN109, and PHE112

^¥^The average of three separate docking runs.

**Fig. 7:**
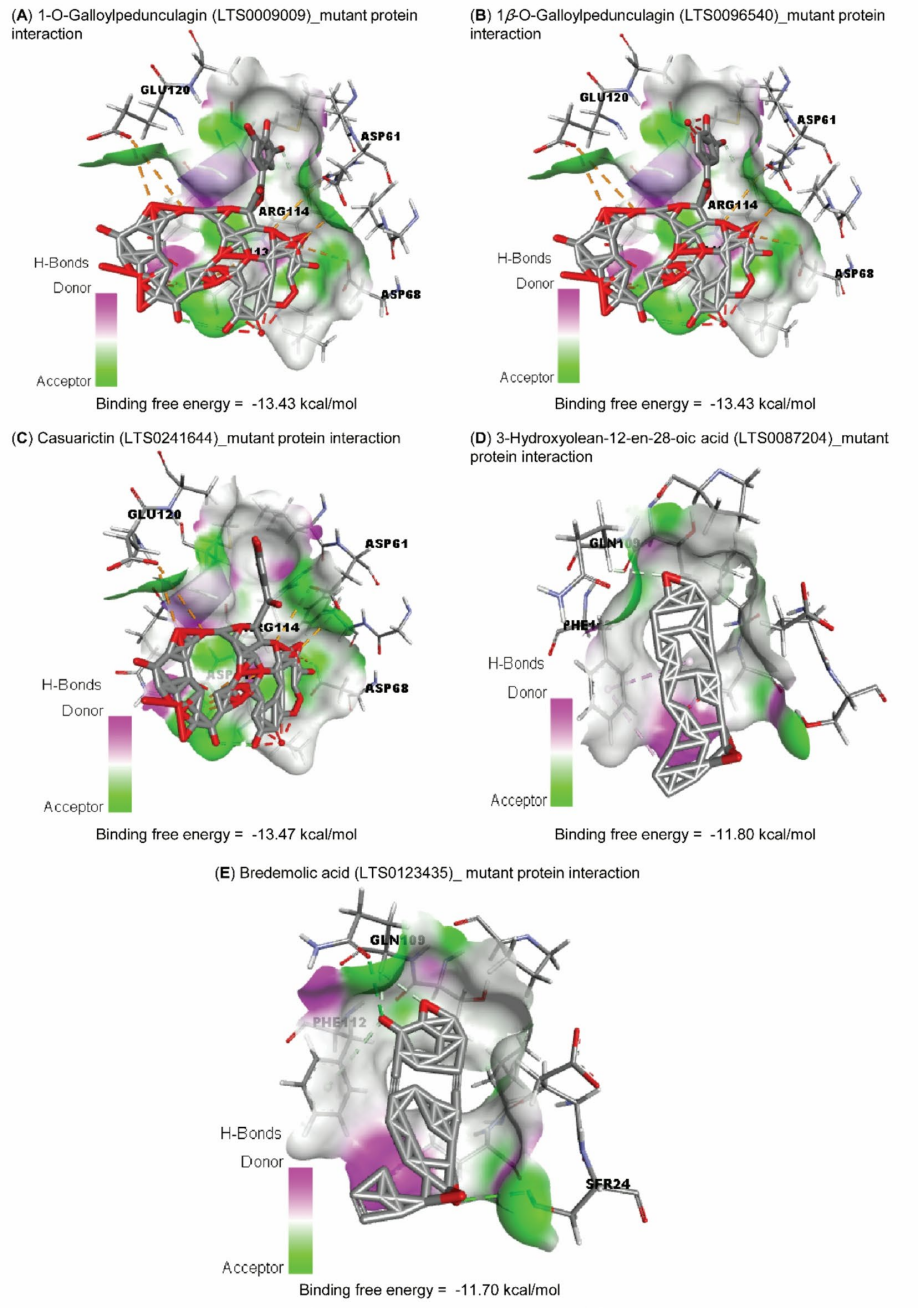
Molecular docking interactions of the top 5 bioactive compounds against the mutant protein. (**A**) 1-O-Galloylpedunculagin (LTS0009009) mutant protein interaction. (**B**) 1*β*-O-Galloylpedunculagin (LTS0096540)_mutant protein interaction. (**C**) Casuarictin (LTS0241644)_mutant protein interaction. (**D**) 3-Hydroxyolean-12-en-28-oic acid (LTS0087204)_mutant protein interaction. (**E**) Bredemolic acid (LTS0123435)_mutant protein interaction.

By docking the same top 5 with the mutant protein, the order of these compounds regarding their free binding energies and binding affinities (pKi) was LTS0009009, LTS0096540, LTS0241644, LTS0087204, and LTS0123435, respectively (Table 4; Fig. 7, **and Supplementary File 2**). These compounds exhibited lower free binding energies towards the mutant protein active site than the wild-type protein.

### Molecular docking of compounds of with 0.99 similarity to LTS0009009, LTS0096540, and LTS0241644

LTS0009009, LTS0096540, and LTS0241644 exhibited higher free binding energy towards the wild-type and mutant proteins, while the compound from LTS0009009 demonstrated higher free binding energy towards the mutant protein. By utilizing ligand similarity prediction through the ChemMine Tools, employing a PubChem fingerprint search with a cutoff of 0.99, we identified 375 similar compounds for LTS0009009, LTS0096540, and LTS0241644 that docked with the wild and mutant proteins, showing free binding energies represented in Supplementary Table 2, Supplementary Tables 3, and Supplementary Table 4, respectively.

### Docking scores of PLRV-RdRp’s wild-type and mutant proteins with RNAs of the initial protein and the putative movement protein of PLRV

Data in **Supplementary Table 5** revealed that RdRp’s mutant protein exhibited higher docking scores toward the RNAs of the PLRV’s initial (−265.029) and putative movement (−260.964) proteins compared to the wild protein (−232.552 and − 239.565, respectively).

## Discussion

The RdRp PCR product was amplified to 593 bp from each isolate, aligning with various host plant species across botanical families and diverse geographic locations. In another study^41^, reported that the generic RT-PCR identified 46 isolates belonging to ten poleroviral species. Nevertheless, to establish a functional replicase, additional host gene products are essential for viral replication, to the P1 and P1P2 proteins^42^.

Numerous studies observed that the phylogenetic identities of the five isolates varied between 96.6% and 98.7%, indicating that, like the sixteen PLRV isolates reported so far (except for one from Australia), there is little sequence variation among the Indian isolates^43^. Earlier papers have distinctly show that isolates from the Netherlands, Scotland, and Canada^44^Egypt^45^Spain, Peru, Cuba, France, Zimbabwe^46^Poland^47^and the Czech Republic^48^ are more similar to each other than those from Australia. Additionally, a phylogenetic tree was constructed using complete genomic nucleotide sequences from thirty isolates in Kenya. These isolates are part of a larger collection of 84 PLRV isolates gathered from 22 countries. A subsequent molecular phylogenetic analysis investigated the evolutionary relationships among all PLRV isolates^49^. In this study, the unrooted tree assessed the relatedness of the DA2 isolate, similar isolates to 17 isolates from ten countries. However, this study found that the most similar isolates to those from Egypt were identified in Germany and India, with the closest sequences coming from Canada and Colombia. The previous findings, combined with the results illustrate that the lines of similarity between studies are quite different. However, some lines have common ancestors, which is evident even among isolates from distant countries.

Jeevalatha et al.^50^ reported that the P0 range of amino acid sequence identity with the other previously reported PLRV isolates was 85.1–100%; for RdRp, it was 96.2–99.6%; for coat protein, it was 96.6–100%; and for movement protein, it was 93.5–100%. Notably, the sequence composition of ordered and disordered proteins was compared in detail, showing that residues known as disorder-promoting residues-Ala, Arg, Gly, Gln, Glu, Lys, Pro, and Ser-occurred more frequently in IDPs/IDRs. Asn, Cys, Ile, Leu, Phe, Val, Trp, and Tyr, on the other hand, were more common in the ordered/structured regions of the proteins (called order-promoting residues)^51–53^. IDPs and regions (IDPRs) engage with many binding partners in protein-protein interactions (PPIs). These unstructured proteins crucially regulate vital functions, including protein complex formation and transcription translation^18,54^. Farooq et al.^49^ found that the protein disorder prediction, CP-RTD, exhibited the highest proportion of disordered amino acids at 48%, compared to CP at 44%, MP at 37%, P1 at 35%, RdRp at 29%, and RTD at 19%. Notably, P0 has the lowest percentage of disordered residues (5.4%). Additionally, RdRp displayed a comparable pattern, with 13% of disordered residues located in the 5′ half and 16.1% in the 3′ end. In another study, Charon et al.^55^ observed that the virus host range may expand because of IDPs’ dynamic engagement in protein-protein interactions. Researchers have demonstrated that IDPs in the PVY genome may facilitate virus adaptation by increasing the exploration of evolutionary pathways or reducing the fitness costs associated with resistance-breaking mutations. Furthermore, IDPs ultimately enhance the ability of RNA viruses to adapt. It is believed that IDRs have a higher mutational permissiveness than highly ordered regions from an evolutionary perspective. Therefore, adaptive solutions may emerge throughout the selection process due to amino acid polymorphisms related to IDRs^56^. It has been observed that the IDPRs change more quickly than the ordered sections of *potyviruses* during their evolutionary history^57^. Notably, another study involving an RNA virus that infects insects (the Nodamura virus, or NoV) demonstrates that rapidly changing IDRs may provide a source of evolutionary innovation and be crucial for adapting viruses to new environments^58^. In this study, the disorder grade prediction of the two dedicated SNPs on the studied isolate and the reference sequence with the wild-type amino acid chain illustrates that the isolate disorder has the highest disorder in the protein function than the reference wild-type. As the studied sequences have 2.5% disorder, but the wild-type sequence reference is 2.1% disorder inSNP (A93T), the disorder’s low probability may come from the fact that they have semi-similar 3D shapes. On the other hand, the studied isolate mutation disorder had a higher disorder in the function of the protein than the reference wild-type, as the studied sequence had 61% disorder, while in the wild-type sequence reference is 13.4% disorder in SNP (K117G), indicating that this SNP has a high disorer effect because lysine has an electrically charged side chain. Still, the glycine has a non-polar side chain. However, these changes did not locate in the active site of the RdRp protein, exhibiting high disorder in function computationally, not experimentally, verification yet. But we suggest that it is considered a good step to identify more of these sites, which help in virus control.

The current study highlights that the identified single nucleotide polymorphisms (SNPs) within the RdRp protein significantly compromises its stability, structural integrity, and interactions with potential therapeutic agents. Therefore, we investigated the molecular interactions of the active ingredients of natural origin in already published studies^37,38^. Notably, compounds extracted from *Syzygium aromaticum*, commonly known as clove, demonstrate a remarkable binding affinity for the RdRp protein. Bredemolic acid shows a strong affinity for the wild-type RdRp, while 1-O-Galloylpedunculagin exhibits preferential binding to the mutant variants of the protein. The selective binding of 1-O-Galloylpedunculagin to the mutant RdRp suggests its potential as a resistance-proof inhibitor, which may guide experimental validation of the compound’s effectiveness against emerging strains. This underscores the need for further investigation into RdRp’s behavior in response to these bioactive compounds and their efficacy in conjunction with viral biocontrol agents.

Additionally, the experimental study conducted by Iftikhar et al.^36^ delves into the potential of utilizing natural herbs, including *Syzygium aromaticum*, for the biocontrol of viral pathogens. Their research suggests that these natural compounds may exhibit significant biocontrol efficacy against the PLRV by targeting the RdRp enzyme, offering promising avenues for sustainable viral management strategies. Generally, the structural destabilization and changes in binding affinity linked to the identified SNPs may arise from their closeness to RdRp’s essential motifs for replication, ultimately destabilizing the RdRp protein. Furthermore, these SNPs altered the binding pockets, potentially accounting for the wild-type’s varying affinity for natural bioactive compounds compared to mutant forms of RdRp. In addition, experimental studies about the effect of these RdRp’ SNPs on PLRV pathogenicity and spreading are urgently needed to find suitable strategies for PLRV biocontrol.

Interestingly, the mutant RdRp protein showed higher docking scores with the RNAs of PLRV’s initial and potential movement proteins than the wild-type protein, resulting in enhanced viral replication and spread. These findings highlight significant differences in how the mutant interacts with viral RNAs, underscoring the need for further investigation to fully comprehend the implications of these changes on viral behavior and pathogenicity. Future studies could investigate the molecular mechanisms behind these interactions and evaluate their potential impact on development targeted antiviral strategies.

## Conclusion

These unidentified processes are likely crucial for PLRV’s adaptability, enhancing its pathogenicity and host range. The findings support the development of rapid diagnostic and sustainable management strategies for PLRV. It is suggested that IDPs and IDPRs help PLRV adaptation by mitigating mutation-related fitness penalties or facilitating unique evolutionary pathways, though further research is needed to verify this. Additionally, SNPs in the RdRp protein negatively impact its stability, structure, physicochemical properties, and binding site interactions by drugs. Bioactive compounds from *S. aromaticum* showed a stronger binding affinity to the RdRp protein, with 1-O-Galloylpedunculagin, 1*β*-O-Galloylpedunculagin, and casuarictin possessing the highest affinities for the wild protein than the mutant protein. Further investigation is needed to assess RdRp proteins’ responsiveness to these compounds as potential biocontrol tools for the virus.

## Supplementary Information

Below is the link to the electronic supplementary material.

Supplementary Material 1

Supplementary Material 2

Supplementary Material 3

Supplementary Material 4

Supplementary Material 5

Supplementary Material 6

Supplementary Material 7

Supplementary Material 8

Supplementary Material 9

## Data Availability

All data generated or analyzed during this study are included in this published article.The datasets analyzed during the current study are available in the UniProt database repository with the ID A0A8E6I3S8 [https://www.uniprot.org/uniprotkb/A0A8E6I3S8/entry]”.
