# Supplementary material for: Prediction of single nucleotide polymorphisms of RNA dependent RNA polymerase for the potato leafroll virus using computational and experimental approaches
Source: Sci Rep. 2025 Aug 17;15:30121. doi: 10.1038/s41598-025-14436-8 (PMC12358528; doi:10.1038/s41598-025-14436-8)
Supplement: Supplementary file 2 — Supplementary Material 2 [file 41598_2025_14436_MOESM2_ESM.pdf]

Prediction of Single Nucleotide Polymorphisms of RNA Dependent RNA Polymerase for the Potato Leafroll Virus Using Computational and Experimental Approaches

```
Seq1_MT576073_          AAAGCCGGATCTGAAGCTGAACTCCAAAGCCTGAATCTACAGGCTGCCAGGTGGCTCCAA
AF453393.1_2073-2464    AAAGCCGGATCTGAAGCTGAACTCCAAAGCCTGAATCTACAGGCTGCCAGGTGGCTCCAA
KC456054.1_2090-2481    AAAGCCGGATCTGAAGCTGAACTCCAAAGCCTGAATCTACAGGCTGCCAGGTGGCTCCAA
AF453390.1_2046-2437    AAAGCCGGATCTGAAGCTGAACTCCAAAGCCTGAATCTACAGGCTGCCAGGTGGCTCCAA
JQ346190.1_2073-2464    AAAGCCGGATCTGAAGCTGAACTCCAAAGCCTGAATCTACAGGCTGCCAGGTGGCTCCAA
MH937415.1_2090-2481    AAAGCCGGATCTGAAGCTGAACTCCAAAGCCTGAATCTACAGGCTGCCAGGTGGCTCCAA
KC456053.1_2090-2481    AAAGCCGGATCTGAAGCTGAACTCCAAAGCCTGAATCTACAGGCTGCCAGGTGGCTCCAA
JQ420902.1_2090-2481    AAAGCCGGATCTGAAGCTGAACTCCAAAGCCTGAATCTACAGGCTGCCAGGTGGCTCCAA
KY856831.1_2089-2480    AAAGCCGGATCTGAAGCTGAACTCCAAAGCCTGAATCTACAGGCTGCCAGGTGGCTCCAA
JQ420905.1_2090-2481    AAAGCCGGATCTGAAGCTGAACTCCAAAGTCTGAATCTACAGGCTGCCAGGTGGCTCCAA
AF453392.1_2073-2464    AAAGCCGGATCTGAAGCTGAACTCCAAAGCCTGAATCTACAGGCTGCCAGGTGGCTCCAA
MN950791.1_2078-2469    AAAGCCGGATCTGAAGCTGAACTCCAAAGCCTGAATCTACAGGCTGCCAGGTGGCTCCAA
KX712226.1_2097-2488    AAAGCCGGATCTGAAGCTGAACTCCAAAGCCTGAATCTACAGGCTGCCAGGTGGCTCCAA
MN694918.1_2078-2469    AAAGCCGGATCTGAAGCTGAGCTCCAAAGCCTGAATCTACAGGCTGCCAGGTGGCTTCAA
                        ******************** ******** ************************** ***

Seq1_MT576073_          CGCGCGGAGTCGGCCACTATCCCTGGCGCAGAAGCAAGAAAGCGCGTGATTGAGAAAACA
AF453393.1_2073-2464    CGCGCGGAGTCGGCCACTATCCCTGGCGCAGAAGCAAGAAAGCGCGTGATTGAGAAAACA
KC456054.1_2090-2481    CGCGCGGAGTCGGCCACTATCCCTGGCGCAGAAGCAAGAAAGCGCGTGATTGAGAAAACA
AF453390.1_2046-2437    CGCGCGGAGTCGGCCACTATCCCTGGCGCAGAAGCAAGAAAGCGCGTGATTGAGAAAACA
JQ346190.1_2073-2464    CGCGCAGAGTCGGCCACTATCCCTGGCGCAGAAGCAAGAAAGCGCGTGATTGAGAAAACA
MH937415.1_2090-2481    CGCGCGGAGTCGGCCACTATCCCTGGCGCAGAAGCAAGAAAGCGCGTGATTGAGAAAACA
KC456053.1_2090-2481    CGCGCGGAGTCGGCCACTATCCCTGGCGCAGAAGCAAGAAAGCGCGTGATTGAGAAAACA
KY856831.1_2089-2480    CGCGCGGAGTCGGCCACTATCCCTGGCGCAGAAGCAAGAAAGCGCGTGATTGAGAAAACA
AF453392.1_2073-2464    CGTGCGGAGTCAGCCACTATCCCTGGCGCAGAAGCAAGAAAGCGCGTGATTGAGAAAACA
MN950791.1_2078-2469    CGCGCGGAGTCAGCCACTATCCCTGGCGCAGAAGCAAGAAAGCGCGTGATCGAGAAAACA
KX712226.1_2097-2488    CGCGCGGGGTCGGCCACTATCCCTGGCGCAGAAGCAAGAAAGCGCGTGATTGAGAAAACA
MN694918.1_2078-2469    CGCGCGGAGTCGGCCACTATCCCTGGCGCAGAAGCAAGAAAGCGCGTGATTGAGAAAACA
                        ** ** * *** ************************************** *********

Seq1_MT576073_          GTGGAGGCATACAGAAATTGTGTAACTAACGCCCCACTGTGCTCCCTTAAATCCAAACTG
AF453393.1_2073-2464    GTGGAGGCATACAGAAATTGTGTAACTAACGCCCCACTGTGCTCCCTTAAATCCAAACTG
KC456054.1_2090-2481    GTGGAGGCATACAGAAATTGTGTAACTAACGCCCCACTGTGCTCCCTTAAATCCAAACTG
AF453390.1_2046-2437    GTGGAGGCATACAGAAATTGTGTAACTAACGCCCCACTGTGCTCCCTTAAATCCAAACTG
JQ346190.1_2073-2464    GTGGAGGCATACAGAAATTGTGTAACTAACGCCCCACTGTGCTCCCTTAAATCCAAACTG
MH937415.1_2090-2481    GTGGAGGCATACAGAAATTGTGTAACTAACGCCCCACTGTGCTCCCTTAAATCCAAACTG
KC456053.1_2090-2481    GTGGAGGCATACAGAAATTGTGTAACTAACGCCCCACTGTGCTCCCTCAAATCCAAACTG
KY856831.1_2089-2480    GTGGAGGCATACAGAAATTGTGTAACTAACGCCCCACTATGCTCCCTTAAATCCAAACTG
AF453392.1_2073-2464    GTGGAGGCATACAGAAATTGTGTAACTAACGCCCCACTATGCTCCCTTAAATCCAAACTG
MN950791.1_2078-2469    GTGGAGGCATACAGAAATTGTGTAACTAACGCCCCACTGTGCTCCCTTAAATCCAAACTG
KX712226.1_2097-2488    GTGGAGGCATACAGAAATTGCGTAACTAACGCCCCACTGTGCTCCCTTAAATCCAAATTG
MN694918.1_2078-2469    GTGGAGACATACAGAAATTGTGTAACTAACGCCCCACTGTGCTCCCTTAAATCCAAACTG
                        ****** **** ******** ***************** ******** ********  **

Seq1_MT576073_          GATTGGGCTGGCTTTCAACAAGATATCCGTGAAGCAGTCCAGTCCCTTGAGCTAGACGCT
AF453393.1_2073-2464    GATTGGGCTGGCTTTCAACAAGATATCCGTGAAGCAGTCCAGTCCCTTGAGCTAGACGCT
KC456054.1_2090-2481    GATTGGGCTGGCTTTCAACAAGATATCCGTGAAGCAGTCCAGTCCCTTGAGCTAGACGCT
AF453390.1_2046-2437    GATTGGGCTGGCTTTCAACAAGATATCCGTGAAGCAGTCCAGTCCCTTGAGCTAGACGCT
JQ346190.1_2073-2464    GATTGGGCTGGCTTTCAACAAGATATTCGTGAAGCAGTCCAGTCCCTTGAGCTAGACGCT
MH937415.1_2090-2481    GATTGGGCTGGCTTTCAACAAGATATCCGTGAAGCAGTCCAGTCCCTTGAGCTAGACGCT
KC456053.1_2090-2481    GATTGGGCTGGCTTTCAACAAGATATCCGTGAAGCAGTCCAGTCCCTTGAGCTAGACGCT
KY856831.1_2089-2480    GATTGGGCTGGCTTTCAACAAGATATCCGTGAAGCAGTCCAGTCCCTTGAGCTAGACGCT
AF453392.1_2073-2464    GATTGGGCTGGCTTTCAACAAGATATCCGTGAAGCAGTCCAGTCCCTTGAGCTAGACGCT
MN950791.1_2078-2469    GATTGGGCTGGCTTTCAACAAGATATCCGTGAAGCAGTCCAGTCCCTTGAGCTAGACGCC
KX712226.1_2097-2488    GATTGGGCTGGCTTTCAACAAGATATCCGTGAAGCAGTCCAGTCCCTTGAGCTAGACGCT
MN694918.1_2078-2469    GATTGGGCTGGCTTTCAACAAGACATCCGTGAAGCAGTCCAGTCCCTTGAGCTAGACGCT
                        *********************** ** ************** ***************** 

Seq1_MT576073_          GGTGTAGGCATTCCCTATATCGCGTATGGCCTCCCCACACACCGAGGATGGGTTGAGGAC
AF453393.1_2073-2464    GGTGTAGGCATTCCCTATATCGCGTATGGCCTCCCCACACACCGAGGATGGGTTGAGGAC
KC456054.1_2090-2481    GGTGTAGGCATTCCCTATATCGCGTATGGCCTCCCCACACACCGAGGATGGGTTGAGGAC
AF453390.1_2046-2437    GGTGTAGGCATTCCCTATATCGCGTATGGCCTCCCCACACACCGAGGATGGGTTGAGGAC
JQ346190.1_2073-2464    GGTGTAGGCATTCCCTATATCGCGTATGGCCTCCCCACACACCGAGGATGGGTTGAGGAC
```

```
MH937415.1_2090-2481       GGTGTAGGCATTCCCTATATCGCGTATGGTCTCCCCACACACCGAGGATGGGTTGAGGAC
KC456053.1_2090-2481       GGTGTAGGCATTCCCTATATCGCGTATGGCCTCCCCACACACCGAGGATGGGTTGAGGAC
KY856831.1_2089-2480       GGTGTAGGCATTCCCTATATCGCGTATGGCCTCCCCACACACCGAGGATGGGTTGAGGAC
AF453392.1_2073-2464       GGTGTAGGCATTCCCTATATAGCGTACGGCCTCCCCACACACCGAGGGTGGGTTGAGGAC
MN950791.1_2078-2469       GGTGTAGGCATTCCCTATATCGCGTATGGCCTCCCCACACACCGAGGATGGGTTGAGGAC
KX712226.1_2097-2488       GGTGTAGGTATTCCCTATATCGCGTATGGCCTCCCCACACACCGAGGATGGGTTGAGGAC
MN694918.1_2078-2469       GGTGTAGGCATTCCCTACATCGCGTATGGCCTCCCCACACACCGAGGATGGGTTGAGGAC
                           ******** ** ***** ** ***** ** ***************** ************

Seq1_MT576073_             CATAAGCTTCTCCCAGTGCTCACTCAGCTGACCTTTGACCGACTACAGGGGATGTCAGAG
AF453393.1_2073-2464       CATAAGCTTCTCCCAGTGCTCACTCAGCTGACCTTTGACCGACTACAGGGGATGTCAGAG
KC456054.1_2090-2481       CATAAGCTTCTCCCAGTGCTCACTCAGCTGACCTTTGACCGACTACAGGGGATGTCAGAG
AF453390.1_2046-2437       CATAAGCTTCTCCCAGTGCTCACTCAGCTGACCTTTGACCGACTACAGGGGATGTCAGAG
JQ346190.1_2073-2464       CATAAGCTTCTCCCAGTGCTCACTCAGCTGACCTTTGACCGACTACAGGGGATGTCAGAG
MH937415.1_2090-2481       CATAAGCTTCTCCCAGTGCTCACTCAGCTGACCTTTGACCGACTACAGGGGATGTCAGAG
KC456053.1_2090-2481       CATAAGCTTCTCCCAGTGCTCACTCAGCTGACCTTTGACCGACTACAGGGGATGTCAGAG
KY856831.1_2089-2480       CATAAGCTTCTCCCAGTGCTCACTCAGCTGACCTTTGACCGACTACAGGGGATGTCAGAG
AF453392.1_2073-2464       CATAAGCTTCTCCCAGTGCTCACTCAGCTGACCTTTGACCGACTACAGAAGATGTCAGAG
MN950791.1_2078-2469       CATAAGCTTCTCCCAGTGCTCACTCAGCAGACCTTTGACCGACTACAGAAGATGTCGGAG
KX712226.1_2097-2488       CATAAGCTTCTCCCAGTACTCACTCAGCTGACCTTTGACCGACTACAGAAGATGTCGGAG
MN694918.1_2078-2469       CATAAGCTTCTCCCAGTGCTCACTCAGCTGACCTTTGACCGACTACAGAAGATGTCGGAG
                           ***************** ********** ************  *****  ****** ***

Seq1_MT576073_             GCCAGCTTTGAGGATATGAGCGCAAGAAGAGCTAAACAAGGA
AF453393.1_2073-2464       GCCAGCTTTGAGGATATGAGCGCA-GAAGAGCT---------
KC456054.1_2090-2481       GCCAGCTTTGAGGATATGAGCGCA-GAAGAGCT---------
AF453390.1_2046-2437       GCCAGCTTTGAGGATATGAGCGCA-GAAGAGCT---------
JQ346190.1_2073-2464       GCCAGCTTTGAGGATATGAGCGCA-GAAGAGCT---------
MH937415.1_2090-2481       GCCAGCTTTGAGGATATGAGCGCA-GAAGAGCT---------
KC456053.1_2090-2481       GCCAGCTTTGAGGATATGAGCGCA-GAAGAGCT---------
KY856831.1_2089-2480       GCCAGCTTTGAGGATATGAGCGCA-GAAGAGCT---------
AF453392.1_2073-2464       GCCAGCTTTGAGGATATGAGCGCA-GAAGAGCT---------
MN950791.1_2078-2469       GCCAGCTTTGAAGATATGAGCGCA-GAAGAGCT---------
KX712226.1_2097-2488       GCCAGCTTTGAGGATATGAGCGCG-GAAGAGCT---------
MN694918.1_2078-2469       GCCAGCTTTGAGGATATGAGCGCA-GAAGAGCT---------
                           *********** ******** **


JQ420903.1_2090-2481        AACTCTGCCAAGTTCATCCCGAGCTGGCGGAGAAAACAGCAGGATTCGGCTGGCCAAAAG
JQ346190.1_2073-2464        AACTCTGCCAAGTTCATCCCGAGCTGGCGGAGAAAACAGCAGAATTCGGCTGGCCAAAAG
MH937415.1_2090-2481        AACTCTGCCAAGTTCATCCCGAGCTGGCGGAGAAAACAGCAGGATTCGGCTGGCCAAAAG
AF453392.1_2073-2464        AACTCTGCCAAGTTCATCCCGAGCTGGCGGAGAAAACAGCAGGATTCGGCTGGCCAAAAG
JQ420904.1_2090-2481        AACTCTGCCAAGTTCATCCCGAGCTGGCGGAGAAAACAGCAGGATTCGGCTGGCCAAAAG
MN694918.1_2078-2469        GACTCTGCCAAGTTCATCCCGAGCTGGCGGAGAAAACAGCAGGATTCGGCTGGCCAAAAG
MN950791.1_2078-2469        AACTCTGCCAAGTTCACCCCGAGCTGGCGGAGAAAACAGCAGGATTCGGCTGGCCAAAAG
JQ420902.1_2090-2481        AACTCTGCCAAGTTCATCCCGAGCTGGCGGAGAAAACAGCAGGATTCGGCTGGCCAAAAG
KC456054.1_2090-2481        AACTCTGCCAAGTTCATTCCGAGCTGGCGGAGAAAACAGCAGGATTCGGCTGGCCAAAAG
KC456053.1_2090-2481        AACTCTGCCAAGTTCATCCCGAGCTGGCGGAGAAAACAGCAGGATTCGGCTGGCCAAAAG
AF453390.1_2046-2437        AACTCTGCCAAGTTCATCCCGAGCTGGCGGAGAAAACAGCAGGATTCGGCTGGCCAAAAG
AF453393.1_2073-2464        GACTCTGCCAAGTTCATCCCGAGCTGGCGGAGAAAACAGCAGGATTCGGCTGGCCAAAAG
Seq1_MT576073_              --------------------------------------------------------AAAG
KY856831.1_2089-2480        AACTCTGCCAAGTTCATCCCGAGCTGGCGGAGAAAACAGCAGGATTCGGCTGGCCAAAAG
KX712226.1_2097-2488        AACTCTGCCAAGTTCATCCCGAGCTGGCGGAGAAAACAGCAGGATTCGGTTGGCCAAAAG
                                                                                    ****

JQ420903.1_2090-2481       CCGGATCTGAAGCTGAACTCCAAAGCCTGAATCTACAGGCTGCCAGGTGGCTCCAACGCG
JQ346190.1_2073-2464       CCGGATCTGAAGCTGAACTCCAAAGCCTGAATCTACAGGCTGCCAGGTGGCTCCAACGCG
MH937415.1_2090-2481       CCGGATCTGAAGCTGAACTCCAAAGCCTGAATCTACAGGCTGCCAGGTGGCTCCAACGCG
AF453392.1_2073-2464       CCGGATCTGAAGCTGAACTCCAAAGCCTGAATCTACAGGCTGCCAGGTGGCTCCAACGTG
JQ420904.1_2090-2481       CCGGATCTGAAGCTGAACTCCAAAGCCTGAATCTACAGGCTGCCAGGTGGCTCCAACGCG
MN694918.1_2078-2469       CCGGATCTGAAGCTGAGCTCCAAAGCCTGAATCTACAGGCTGCCAGGTGGCTTCAACGCG
MN950791.1_2078-2469       CCGGATCTGAAGCTGAACTCCAAAGCCTGAATCTACAGGCTGCCAGGTGGCTCCAACGCG
JQ420902.1_2090-2481       CCGGATCTGAAGCTGAACTCCAAAGCCTGAATCTACAGGCTGCCAGGTGGCTCCAACGCG
KC456054.1_2090-2481       CCGGATCTGAAGCTGAACTCCAAAGCCTGAATCTACAGGCTGCCAGGTGGCTCCAACGCG
KC456053.1_2090-2481       CCGGATCTGAAGCTGAACTCCAAAGCCTGAATCTACAGGCTGCCAGGTGGCTCCAACGCG
AF453390.1_2046-2437       CCGGATCTGAAGCTGAACTCCAAAGCCTGAATCTACAGGCTGCCAGGTGGCTCCAACGCG
AF453393.1_2073-2464       CCGGATCTGAAGCTGAACTCCAAAGCCTGAATCTACAGGCTGCCAGGTGGCTCCAACGCG
Seq1_MT576073_             CCGGATCTGAAGCTGAACTCCAAAGCCTGAATCTACAGGCTGCCAGGTGGCTCCAACGCG
KY856831.1_2089-2480       CCGGATCTGAAGCTGAACTCCAAAGCCTGAATCTACAGGCTGCCAGGTGGCTCCAACGCG
KX712226.1_2097-2488       CCGGATCTGAAGCTGAACTCCAAAGCCTGAATCTACAGGCTGCCAGGTGGCTCCAACGCG
                           **************** *********************************** ***** *

JQ420903.1_2090-2481       CGGAGTCGGCCACTATCCCTGGCGCAGAAGCAAGAAAGCGCGTGATTGAGAAAACAGTGG
JQ346190.1_2073-2464       CAGAGTCGGCCACTATCCCTGGCGCAGAAGCAAGAAAGCGCGTGATTGAGAAAACAGTGG
MH937415.1_2090-2481       CGGAGTCGGCCACTATCCCTGGCGCAGAAGCAAGAAAGCGCGTGATTGAGAAAACAGTGG
AF453392.1_2073-2464       CGGAGTCAGCCACTATCCCTGGCGCAGAAGCAAGAAAGCGCGTGATTGAGAAAACAGTGG
JQ420904.1_2090-2481       CGGAGTCGGCCACTATCCCTGGCGCAGAAGCAAGAAAGCGCGTGATTGAGAAAACAGTGG
MN694918.1_2078-2469       CGGAGTCGGCCACTATCCCTGGCGCAGAAGCAAGAAAGCGCGTGATTGAGAAAACAGTGG
MN950791.1_2078-2469       CGGAGTCAGCCACTATCCCTGGCGCAGAAGCAAGAAAGCGCGTGATCGAGAAAACAGTGG
JQ420902.1_2090-2481       CGGAGTCGGCCACTATCCCTGGCGCAGAAGCAAGAAAGCGCGTGATTGAGAAAACAGTGG
KC456054.1_2090-2481       CGGAGTCGGCCACTATCCCTGGCGCAGAAGCAAGAAAGCGCGTGATTGAGAAAACAGTGG
KC456053.1_2090-2481       CGGAGTCGGCCACTATCCCTGGCGCAGAAGCAAGAAAGCGCGTGATTGAGAAAACAGTGG
```

```
AF453390.1_2046-2437      CGGAGTCGGCCACTATCCCTGGCGCAGAAGCAAGAAAGCGCGTGATTGAGAAAACAGTGG
AF453393.1_2073-2464      CGGAGTCGGCCACTATCCCTGGCGCAGAAGCAAGAAAGCGCGTGATTGAGAAAACAGTGG
Seq1_MT576073_            CGGAGTCGGCCACTATCCCTGGCGCAGAAGCAAGAAAGCGCGTGATTGAGAAAACAGTGG
KY856831.1_2089-2480      CGGAGTCGGCCACTATCCCTGGCGCAGAAGCAAGAAAGCGCGTGATTGAGAAAACAGTGG
KX712226.1_2097-2488      CGGGGTCGGCCACTATCCCTGGCGCAGAAGCAAGAAAGCGCGTGATTGAGAAAACAGTGG
                          * * *** ************************************** *************

JQ420903.1_2090-2481      AGGCATACAGAAATTGTGTAACTAACGCCCCACTGTGCTCCCTTAAATCCAAACTGGATT
JQ346190.1_2073-2464      AGGCATACAGAAATTGTGTAACTAACGCCCCACTGTGCTCCCTTAAATCCAAACTGGATT
MH937415.1_2090-2481      AGGCATACAGAAATTGTGTAACTAACGCCCCACTGTGCTCCCTTAAATCCAAACTGGATT
AF453392.1_2073-2464      AGGCATACAGAAATTGTGTAACTAACGCCCCACTATGCTCCCTTAAATCCAAACTGGATT
JQ420904.1_2090-2481      AGGCATACAGAAATTGTGTAACTAACGCCCCACTGTGCTCCCTTAAATCCAAGCTGGATT
MN694918.1_2078-2469      AGACATACAGAAATTGTGTAACTAACGCCCCACTGTGCTCCCTTAAATCCAAACTGGATT
MN950791.1_2078-2469      AGGCATACAGAAATTGTGTAACTAACGCCCCACTGTGCTCCCTTAAATCCAAACTGGATT
JQ420902.1_2090-2481      AGGCATACAGAAATTGTGTAACTAACGCCCCACTGTGCTCCCTTAAATCCAAACTGGATT
KC456054.1_2090-2481      AGGCATACAGAAATTGTGTAACTAACGCCCCACTGTGCTCCCTTAAATCCAAACTGGATT
KC456053.1_2090-2481      AGGCATACAGAAATTGTGTAACTAACGCCCCACTGTGCTCCCTCAAATCCAAACTGGATT
AF453390.1_2046-2437      AGGCATACAGAAATTGTGTAACTAACGCCCCACTGTGCTCCCTTAAATCCAAACTGGATT
AF453393.1_2073-2464      AGGCATACAGAAATTGTGTAACTAACGCCCCACTGTGCTCCCTTAAATCCAAACTGGATT
Seq1_MT576073_            AGGCATACAGAAATTGTGTAACTAACGCCCCACTGTGCTCCCTTAAATCCAAACTGGATT
KY856831.1_2089-2480      AGGCATACAGAAATTGTGTAACTAACGCCCCACTATGCTCCCTTAAATCCAAACTGGATT
KX712226.1_2097-2488      AGGCATACAGAAATTGCGTAACTAACGCCCCACTGTGCTCCCTTAAATCCAAATTGGATT
                          ** ************* ***************** ******** ********  ******

JQ420903.1_2090-2481      GGGCTGGCTTTCAACAAGATATCCGTGAAGCAGTCCAGTCCCTTGAGCTAGACGCTGGTG
JQ346190.1_2073-2464      GGGCTGGCTTTCAACAAGATATTCGTGAAGCAGTCCAGTCCCTTGAGCTAGACGCTGGTG
MH937415.1_2090-2481      GGGCTGGCTTTCAACAAGATATCCGTGAAGCAGTCCAGTCCCTTGAGCTAGACGCTGGTG
AF453392.1_2073-2464      GGGCTGGCTTTCAACAAGATATCCGTGAAGCAGTCCAGTCCCTTGAGCTAGACGCTGGTG
JQ420904.1_2090-2481      GGGCTGGCTTTCAACAAGATATTCGTGAAGCAGTCCAGTCCCTTGAGCTAGACGCTGGTG
MN694918.1_2078-2469      GGGCTGGCTTTCAACAAGACATCCGTGAAGCAGTCCAGTCCCTTGAGCTAGACGCTGGTG
MN950791.1_2078-2469      GGGCTGGCTTTCAACAAGATATCCGTGAAGCAGTCCAGTCCCTTGAGCTAGACGCCGGTG
JQ420902.1_2090-2481      GGGCTGGCTTTCAACAAGATATCCGTGAAGCAGTCCAGTCCCTTGAGCTAGACGCTGGTG
KC456054.1_2090-2481      GGGCTGGCTTTCAACAAGATATCCGTGAAGCAGTCCAGTCCCTTGAGCTAGACGCTGGTG
KC456053.1_2090-2481      GGGCTGGCTTTCAACAAGATATCCGTGAAGCAGTCCAGTCCCTTGAGCTAGACGCTGGTG
AF453390.1_2046-2437      GGGCTGGCTTTCAACAAGATATCCGTGAAGCAGTCCAGTCCCTTGAGCTAGACGCTGGTG
AF453393.1_2073-2464      GGGCTGGCTTTCAACAAGATATCCGTGAAGCAGTCCAGTCCCTTGAGCTAGACGCTGGTG
Seq1_MT576073_            GGGCTGGCTTTCAACAAGATATCCGTGAAGCAGTCCAGTCCCTTGAGCTAGACGCTGGTG
KY856831.1_2089-2480      GGGCTGGCTTTCAACAAGATATCCGTGAAGCAGTCCAGTCCCTTGAGCTAGACGCTGGTG
KX712226.1_2097-2488      GGGCTGGCTTTCAACAAGATATCCGTGAAGCAGTCCAGTCCCTTGAGCTAGACGCTGGTG
                          ******************* ** ******************************** ****

JQ420903.1_2090-2481      TAGGCATTCCCTATATCGCGTATGGCCTCCCCACACACCGAGGATGGGTTGAGGACCATA
JQ346190.1_2073-2464      TAGGCATTCCCTATATCGCGTATGGCCTCCCCACACACCGAGGATGGGTTGAGGACCATA
MH937415.1_2090-2481      TAGGCATTCCCTATATCGCGTATGGTCTCCCCACACACCGAGGATGGGTTGAGGACCATA
AF453392.1_2073-2464      TAGGCATTCCCTATATAGCGTACGGCCTCCCCACACACCGAGGGTGGGTTGAGGACCATA
JQ420904.1_2090-2481      TAGGCATTCCCTATATCGCGTATGGCCTCCCCACACACCGAGGATGGGTTGAGGACCATA
MN694918.1_2078-2469      TAGGCATTCCCTACATCGCGTATGGCCTCCCCACACACCGAGGATGGGTTGAGGACCATA
MN950791.1_2078-2469      TAGGCATTCCCTATATCGCGTATGGCCTCCCCACACACCGAGGATGGGTTGAGGACCATA
JQ420902.1_2090-2481      TAGGCATTCCCTATATCGCGTATGGCCTCCCCACACACCGAGGATGGGTTGAGGACCATA
KC456054.1_2090-2481      TAGGCATTCCCTATATCGCGTATGGCCTCCCCACACACCGAGGATGGGTTGAGGACCATA
KC456053.1_2090-2481      TAGGCATTCCCTATATCGCGTATGGCCTCCCCACACACCGAGGATGGGTTGAGGACCATA
AF453390.1_2046-2437      TAGGCATTCCCTATATCGCGTATGGCCTCCCCACACACCGAGGATGGGTTGAGGACCATA
AF453393.1_2073-2464      TAGGCATTCCCTATATCGCGTATGGCCTCCCCACACACCGAGGATGGGTTGAGGACCATA
Seq1_MT576073_            TAGGCATTCCCTATATCGCGTATGGCCTCCCCACACACCGAGGATGGGTTGAGGACCATA
KY856831.1_2089-2480      TAGGCATTCCCTATATCGCGTATGGCCTCCCCACACACCGAGGATGGGTTGAGGACCATA
KX712226.1_2097-2488      TAGGTATTCCCTATATCGCGTATGGCCTCCCCACACACCGAGGATGGGTTGAGGACCATA
                          **** ******** ** ***** ** ***************** ****************

JQ420903.1_2090-2481      AGCTTCTCCCAGTGCTCACTCAGCTGACCTTTGACCGACTACAGGGGATGTCAGAGGCCA
JQ346190.1_2073-2464      AGCTTCTCCCAGTGCTCACTCAGCTGACCTTTGACCGACTACAGGGGATGTCAGAGGCCA
MH937415.1_2090-2481      AGCTTCTCCCAGTGCTCACTCAGCTGACCTTTGACCGACTACAGGGGATGTCAGAGGCCA
AF453392.1_2073-2464      AGCTTCTCCCAGTGCTCACTCAGCTGACCTTTGACCGACTACAGAAGATGTCAGAGGCCA
JQ420904.1_2090-2481      AGCTTCTCCCAGTGCTCACTCAGCTGACCTTTGACCGACTACAGGGGATGTCAGAGGCCA
MN694918.1_2078-2469      AGCTTCTCCCAGTGCTCACTCAGCTGACCTTTGACCGACTACAGAAGATGTCGGAGGCCA
MN950791.1_2078-2469      AGCTTCTCCCAGTGCTCACTCAGCAGACCTTTGACCGACTACAGAAGATGTCGGAGGCCA
JQ420902.1_2090-2481      AGCTTCTCCCAGTGCTCACTCAGCTGACCTTTGACCGACTACAGGGGATGTCAGAGGCCA
KC456054.1_2090-2481      AGCTTCTCCCAGTGCTCACTCAGCTGACCTTTGACCGACTACAGGGGATGTCAGAGGCCA
KC456053.1_2090-2481      AGCTTCTCCCAGTGCTCACTCAGCTGACCTTTGACCGACTACAGGGGATGTCAGAGGCCA
AF453390.1_2046-2437      AGCTTCTCCCAGTGCTCACTCAGCTGACCTTTGACCGACTACAGGGGATGTCAGAGGCCA
AF453393.1_2073-2464      AGCTTCTCCCAGTGCTCACTCAGCTGACCTTTGACCGACTACAGGGGATGTCAGAGGCCA
Seq1_MT576073_            AGCTTCTCCCAGTGCTCACTCAGCTGACCTTTGACCGACTACAGGGGATGTCAGAGGCCA
KY856831.1_2089-2480      AGCTTCTCCCAGTGCTCACTCAGCTGACCTTTGACCGACTACAGGGGATGTCAGAGGCCA
KX712226.1_2097-2488      AGCTTCTCCCAGTACTCACTCAGCTGACCTTTGACCGACTACAGAAGATGTCGGAGGCCA
                          ************* ********** *******************  ****** *******

JQ420903.1_2090-2481      GCTTTGAGGATATGAGCGCA-GAAGAGCTGGTTCAAGAAGGGCTCTGTGATCCTATCAGA
JQ346190.1_2073-2464      GCTTTGAGGATATGAGCGCA-GAAGAGCTGGTTCAAGAAGGGCTCTGTGATCCTATCAGA
MH937415.1_2090-2481      GCTTTGAGGATATGAGCGCA-GAAGAGCTGGTTCAAGAAGGGCTCTGTGATCCTATCAGA
AF453392.1_2073-2464      GCTTTGAGGATATGAGCGCA-GAAGAGCTGGTTCAAGAGGGGCTCTGTGATCCTATCAGA
JQ420904.1_2090-2481      GCTTTGAGGATATGAGCGCA-GAAGAGCTGGTTCAAGAAGGGCTCTGTGATCCTATCAGA
MN694918.1_2078-2469      GCTTTGAGGATATGAGCGCA-GAAGAGCTGGTTCAAGAAGGGCTCTGTGATCCTATCAGA
```

```
MN950791.1_2078-2469      GCTTTGAAGATATGAGCGCA-GAAGAGCTGGTTCAAGAAGGGCTCTGTGATCCTATCAGA
JQ420902.1_2090-2481      GCTTTGAGGATATGAGTGCA-GAAGAGCTGGTTCAAGAAGGGCTCTGTGATCCTATCAGA
KC456054.1_2090-2481      GCTTTGAGGATATGAGCGCA-GAAGAGCTGGTTCAAGAAGGGCTCTGTGATCCTATCAGA
KC456053.1_2090-2481      GCTTTGAGGATATGAGCGCA-GAAGAGCTGGTTCAAGAAGGGCTCTGTGATCCTATCAGA
AF453390.1_2046-2437      GCTTTGAGGATATGAGCGCA-GAAGAGCTGGTTCAAGAAGGGCTTTGTGATCCTATCAGA
AF453393.1_2073-2464      GCTTTGAGGATATGAGCGCA-GAAGAGCTGGTTCAAGAAGGGCTCTGTGACCCTATCAGA
Seq1_MT576073_            GCTTTGAGGATATGAGCGCAAGAAGAGCTAAACAAGGA-----------------------
KY856831.1_2089-2480      GCTTTGAGGATATGAGCGCA-GAAGAGCTGGTTCAAGAAGGGCTCTGTGATCCTATCAGA
KX712226.1_2097-2488      GCTTTGAGGATATGAGCGCG-GAAGAGCTGGTTCAAGAAGGGCTCTGTGATCCTATCAGA
                          ******* ******** **  ********      * **
```

Fig. S1. PLRV-RdRp sequence of our Egyptian isolate compared with reference sequences from nine distinct regions.
