# Supplementary material for: Prediction of single nucleotide polymorphisms of RNA dependent RNA polymerase for the potato leafroll virus using computational and experimental approaches
Source: Sci Rep. 2025 Aug 17;15:30121. doi: 10.1038/s41598-025-14436-8 (PMC12358528; doi:10.1038/s41598-025-14436-8)
Supplement: Supplementary file 5 — Supplementary Material 5 [file 41598_2025_14436_MOESM5_ESM.pdf]

Prediction of Single Nucleotide Polymorphisms of RNA Dependent RNA Polymerase for the Potato Leafroll Virus Using Computational and Experimental Approaches

Targets	Ligands	Docking Scores										
		**Model_1**	**Model_2**	**Model_3**	**Model_4**	**Model_5**	**Model_6**	**Model_7**	**Model_8**	**Model_9**	**Model_10**	**Averages**
**RNA of Initial protein**	**Wild-type**	-239.95	-236.11	-234.96	-234.60	-233.28	-231.85	-230.67	-229.10	-228.60	-226.40	-232.552
	**Mutant**	-276.29	-270.00	-268.94	-267.41	-265.93	-263.15	-262.83	-262.61	-257.95	-255.18	-265.029
**RNA of putative movement protein**	**Wild- type**	-244.98	-244.98	-244.42	-242.49	-241.35	-241.22	-238.98	-234.43	-231.65	-231.15	-239.565
	**Mutant**	-273.93	-272.04	-262.16	-261.18	-259.95	-259.53	-256.04	-255.59	-255.42	-253.80	-260.964

Supplementary Table 5. Molecular docking scores of RNA-Dependent RNA Polymerase(RdRp)'s wild-type and mutant proteins against RNAs of the initial protein and the putative movement protein of potato leafroll virus (PLRV)
