# Supplementary material for: Prediction of single nucleotide polymorphisms of RNA dependent RNA polymerase for the potato leafroll virus using computational and experimental approaches
Source: Sci Rep. 2025 Aug 17;15:30121. doi: 10.1038/s41598-025-14436-8 (PMC12358528; doi:10.1038/s41598-025-14436-8)
Supplement: Supplementary file 6 — Supplementary Material 6 [file 41598_2025_14436_MOESM6_ESM.pdf]

Prediction of Single Nucleotide Polymorphisms of RNA Dependent RNA Polymerase for the Potato Leafroll Virus Using Computational and Experimental Approaches

	Wild-type protein		Mutant protein	
**ligands (PubChem ID)**	**Binding Free Energy (kcal/mol)**	**pKi**	**Binding Free Energy (kcal/mol)**	**pKi**
**243**	-5.80	4.25	-5.40	3.96
**2345**	-6.20	4.55	-6.20	4.55
**2912**	-6.60	4.84	-6.30	4.62
**3314**	-7.00	5.13	-5.60	4.11
**6758**	-7.20	5.28	-6.50	4.77
**7005**	-6.40	4.69	-6.00	4.40
**7017**	-6.20	4.55	-5.80	4.25
**7456**	-5.90	4.33	-5.20	3.81
**40326**	-6.00	4.40	-6.70	4.91
**40585**	-6.80	4.99	-5.60	4.11

Supplementary Table 4. Molecular docking interactions of compounds with 0.99 similarity to the Casuarictin (LTS0241644) bioactive compound against wild-type and mutant proteins.
