# Supplementary material for: Prediction of single nucleotide polymorphisms of RNA dependent RNA polymerase for the potato leafroll virus using computational and experimental approaches
Source: Sci Rep. 2025 Aug 17;15:30121. doi: 10.1038/s41598-025-14436-8 (PMC12358528; doi:10.1038/s41598-025-14436-8)
Supplement: Supplementary file 7 — Supplementary Material 7 [file 41598_2025_14436_MOESM7_ESM.pdf]

Prediction of Single Nucleotide Polymorphisms of RNA Dependent RNA Polymerase for the Potato Leafroll Virus Using Computational and Experimental Approaches

	Wild-type protein		Mutant protein	
**ligands (PubChem ID)**	**Binding Free Energy (kcal/mol)**	**pKi**	**Binding Free Energy (kcal/mol)**	**pKi**
**73644**	-9.30	6.82	-9.40	6.89
**158198**	-8.80	6.45	-8.70	6.38
**176131**	-8.50	6.23	-8.90	6.53
**452242**	-9.30	6.82	-9.40	6.89
**5315734**	-9.30	6.82	-9.40	6.89
**10533860**	-8.80	6.45	-8.70	6.38
**10557787**	-8.40	6.16	-8.50	6.23
**13932607**	-9.30	6.82	-9.40	6.89
**13932608**	-9.10	6.67	-9.40	6.89
**14284541**	-8.40	6.16	-8.50	6.23
**21669956**	-8.20	6.01	-8.70	6.38
**22524646**	-9.30	6.82	-9.40	6.89
**44303138**	-9.30	6.82	-9.40	6.89
**44567212**	-9.10	6.67	-9.40	6.89
**50990634**	-9.30	6.82	-9.40	6.89
**71722519**	-9.10	6.67	-8.90	6.53
**118701062**	-9.30	6.82	-9.40	6.89
**157579212**	-8.70	6.38	-8.70	6.38
**162986950**	-8.50	6.23	-8.50	6.23
**162986951**	-8.40	6.16	-8.50	6.23
**164771744**	-9.30	6.82	-9.40	6.89

Supplementary Table 2. Molecular docking interactions of compounds with 0.99 similarity to the 1-O-Galloylpedunculagin (LTS0009009) bioactive compound against wild-type and mutant proteins.
