# Supplementary material for: Prediction of single nucleotide polymorphisms of RNA dependent RNA polymerase for the potato leafroll virus using computational and experimental approaches
Source: Sci Rep. 2025 Aug 17;15:30121. doi: 10.1038/s41598-025-14436-8 (PMC12358528; doi:10.1038/s41598-025-14436-8)
Supplement: Supplementary file 8 — Supplementary Material 8 [file 41598_2025_14436_MOESM8_ESM.pdf]

Prediction of Single Nucleotide Polymorphisms of RNA Dependent RNA Polymerase for the Potato Leafroll Virus Using Computational and Experimental Approaches

	Wild-type protein		Mutant protein	
**ligands (PubChem ID)**	**Binding Free Energy (kcal/mol)**	**pKi**	**Binding Free Energy (kcal/mol)**	**pKi**
**243**	-5.80	4.25	-5.40	3.96
**2345**	-8.10	5.94	-5.50	4.03
**2912**	-6.90	5.06	-5.80	4.25
**3314**	-5.10	3.74	-5.80	4.25
**6758**	-7.10	5.21	-6.60	4.84
**7005**	-6.40	4.69	-6.00	4.40
**7017**	-6.20	4.55	-6.80	4.99
**7456**	-5.60	4.11	-5.20	3.81
**40326**	-6.50	4.77	-6.30	4.62
**40585**	-6.80	4.99	-6.40	4.69

Supplementary Table 3. Molecular docking interactions of compounds with 0.99 similarity to the 1*β*-O-Galloylpedunculagin (LTS0096540) bioactive compound against wild-type and mutant proteins.
