# Supplementary material for: Prediction of single nucleotide polymorphisms of RNA dependent RNA polymerase for the potato leafroll virus using computational and experimental approaches
Source: Sci Rep. 2025 Aug 17;15:30121. doi: 10.1038/s41598-025-14436-8 (PMC12358528; doi:10.1038/s41598-025-14436-8)
Supplement: Supplementary file 9 — Supplementary Material 9 [file 41598_2025_14436_MOESM9_ESM.pdf]

Prediction of Single Nucleotide Polymorphisms of RNA Dependent RNA Polymerase for the Potato Leafroll Virus Using Computational and Experimental Approaches

Sources	Ligands (LOTUS ID)	Binding Free Energy (kcal/mol)	pKi
**Aloe vera**	LTS0069982	-10.70	7.85
	LTS0224734	-10.60	7.77
	LTS0101353	-10.40	7.63
	LTS0036808	-10.20	7.48
	LTS0256439	-10.10	7.41
	LTS0098857	-9.90	7.26
	LTS0229992	-9.70	7.11
	LTS0223661	-9.20	6.75
	LTS0070627	-9.20	6.75
	LTS0010619	-9.20	6.75
	LTS0211986	-9.20	6.75
	LTS0197525	-8.90	6.53
	LTS0039598	-8.80	6.45
	LTS0112501	-8.80	6.45
	LTS0153582	-8.80	6.45
	LTS0267513	-8.70	6.38
	LTS0159879	-8.50	6.23
	LTS0126973	-8.40	6.16
	LTS0037040	-8.40	6.16
	LTS0158828	-8.40	6.16
	LTS0250499	-8.30	6.09
	LTS0029105	-8.30	6.09
	LTS0143834	-8.30	6.09
	LTS0031006	-8.20	6.01
	LTS0119475	-8.20	6.01
	LTS0197016	-8.20	6.01
	LTS0228709	-8.20	6.01
	LTS0274534	-8.20	6.01
	LTS0275716	-8.20	6.01
	LTS0083071	-8.10	5.94
	LTS0170582	-8.10	5.94
	LTS0194466	-8.10	5.94

Prediction of Single Nucleotide Polymorphisms of RNA Dependent RNA Polymerase for the Potato Leafroll Virus Using Computational and Experimental Approaches

Sources	Ligands (LOTUS ID)	Binding Free Energy (kcal/mol)	pKi
	LTS0157592	-8.10	5.94
	LTS0219322	-8.00	5.87
	LTS0029311	-8.00	5.87
	LTS0104348	-8.00	5.87
	LTS0200257	-8.00	5.87
	LTS0101529	-7.90	5.79
	LTS0197988	-7.90	5.79
	LTS0154037	-7.90	5.79
	LTS0201798	-7.90	5.79
	LTS0210130	-7.90	5.79
	LTS0256815	-7.90	5.79
	LTS0001902	-7.80	5.72
	LTS0110311	-7.80	5.72
	LTS0217039	-7.70	5.65
	LTS0270933	-7.70	5.65
	LTS0144287	-7.70	5.65
	LTS0168247	-7.70	5.65
	LTS0116396	-7.70	5.65
	LTS0130240	-7.70	5.65
	LTS0249925	-7.70	5.65
	LTS0032099	-7.60	5.57
	LTS0142692	-7.60	5.57
	LTS0178320	-7.60	5.57
	LTS0084414	-7.60	5.57
	LTS0103398	-7.60	5.57
	LTS0113685	-7.60	5.57
	LTS0268777	-7.50	5.50
	LTS0028978	-7.50	5.50
	LTS0241153	-7.50	5.50
	LTS0102304	-7.40	5.43
	LTS0273425	-7.40	5.43
	LTS0004111	-7.40	5.43

Prediction of Single Nucleotide Polymorphisms of RNA Dependent RNA Polymerase for the Potato Leafroll Virus Using Computational and Experimental Approaches

Sources	Ligands (LOTUS ID)	Binding Free Energy (kcal/mol)	pKi
	LTS0200784	-7.30	5.35
	LTS0071224	-7.30	5.35
	LTS0204616	-7.30	5.35
	LTS0269979	-7.30	5.35
	LTS0009883	-7.20	5.28
	LTS0014529	-7.20	5.28
	LTS0055148	-7.20	5.28
	LTS0122106	-7.20	5.28
	LTS0179389	-7.20	5.28
	LTS0189480	-7.20	5.28
	LTS0199217	-7.20	5.28
	LTS0045120	-7.10	5.21
	LTS0172417	-7.10	5.21
	LTS0214267	-7.00	5.13
	LTS0147074	-7.00	5.13
	LTS0049714	-6.90	5.06
	LTS0045627	-6.70	4.91
	LTS0031255	-6.70	4.91
	LTS0168468	-6.70	4.91
	LTS0143764	-6.60	4.84
	LTS0209859	-6.60	4.84
	LTS0224811	-6.60	4.84
	LTS0146973	-6.60	4.84
	LTS0176064	-6.60	4.84
	LTS0136729	-6.60	4.84
	LTS0076474	-6.50	4.77
	LTS0256168	-6.40	4.69
	LTS0163480	-6.30	4.62
	LTS0160968	-6.10	4.47
	LTS0125187	-6.10	4.47
	LTS0024203	-5.90	4.33
	LTS0069518	-5.80	4.25

Prediction of Single Nucleotide Polymorphisms of RNA Dependent RNA Polymerase for the Potato Leafroll Virus Using Computational and Experimental Approaches

Sources	Ligands (LOTUS ID)	Binding Free Energy (kcal/mol)	pKi
	LTS0002248	-5.60	4.11
	LTS0237369	-5.50	4.03
	LTS0262158	-5.40	3.96
	LTS0009878	-5.40	3.96
	LTS0128796	-5.40	3.96
	LTS0241114	-5.30	3.89
	LTS0108205	-5.20	3.81
	LTS0030533	-5.10	3.74
	LTS0105121	-5.10	3.74
	LTS0123172	-5.10	3.74
	LTS0216520	-5.00	3.67
	LTS0013597	-4.80	3.52
	LTS0082229	-4.70	3.45
	LTS0256910	-4.60	3.37
	LTS0217707	-4.00	2.93
	LTS0254684	-3.60	2.64
	LTS0236573	-13.40	9.83
	LTS0070691	-13.00	9.53
	LTS0075454	-13.00	9.53
	LTS0187528	-12.70	9.31
	LTS0032403	-11.00	8.07
	LTS0200644	-11.00	8.07
	LTS0109177	-10.70	7.85
	LTS0184841	-10.50	7.70
	LTS0268841	-10.50	7.70
	LTS0008665	-10.40	7.63
	LTS0257392	-10.40	7.63
	LTS0265485	-10.30	7.55
	LTS0028259	-10.20	7.48
	LTS0082465	-10.20	7.48
	LTS0269880	-9.90	7.26
	LTS0079352	-9.90	7.26

Sources	Ligands (LOTUS ID)	Binding Free Energy (kcal/mol)	pKi
	LTS0141508	-9.90	7.26
	LTS0209810	-9.90	7.26
	LTS0169099	-9.70	7.11
	LTS0013387	-9.70	7.11
	LTS0201694	-9.70	7.11
	LTS0198024	-9.60	7.04
	LTS0193415	-9.40	6.89
	LTS0135598	-9.40	6.89
	LTS0086201	-9.40	6.89
	LTS0189969	-9.40	6.89
	LTS0216416	-9.30	6.82
	LTS0080601	-9.20	6.75
	LTS0055078	-9.20	6.75
	LTS0146305	-9.20	6.75
	LTS0222014	-9.00	6.60
	LTS0100879	-8.90	6.53
	LTS0266836	-8.80	6.45
	LTS0096073	-8.70	6.38
	LTS0091905	-8.60	6.31
	LTS0117550	-8.50	6.23
	LTS0132416	-8.50	6.23
	LTS0023578	-8.50	6.23
	LTS0267242	-8.30	6.09
	LTS0070367	-8.20	6.01
	LTS0231171	-8.20	6.01
	LTS0020678	-8.10	5.94
	LTS0270078	-8.10	5.94
	LTS0236981	-8.10	5.94
	LTS0238565	-8.00	5.87
	LTS0001399	-7.80	5.72
	LTS0056105	-7.80	5.72
	LTS0224133	-7.70	5.65

Sources	Ligands (LOTUS ID)	Binding Free Energy (kcal/mol)	pKi
	LTS0005886	-7.60	5.57
	LTS0158119	-7.60	5.57
	LTS0193112	-7.60	5.57
	LTS0197087	-7.60	5.57
	LTS0203280	-7.60	5.57
	LTS0191235	-7.60	5.57
	LTS0256647	-7.50	5.50
	LTS0263789	-7.30	5.35
	LTS0210705	-7.20	5.28
	LTS0156187	-7.00	5.13
	LTS0056933	-7.00	5.13
	LTS0177102	-6.90	5.06
	LTS0115731	-6.90	5.06
	LTS0176037	-6.90	5.06
	LTS0192119	-6.90	5.06
	LTS0172915	-6.80	4.99
	LTS0041646	-6.80	4.99
	LTS0110530	-6.80	4.99
	LTS0133156	-6.80	4.99
	LTS0136885	-6.80	4.99
	LTS0046643	-6.70	4.91
	LTS0148949	-6.70	4.91
	LTS0162316	-6.70	4.91
	LTS0072900	-6.70	4.91
	LTS0163350	-6.70	4.91
	LTS0259993	-6.70	4.91
	LTS0200483	-6.70	4.91
	LTS0118590	-6.60	4.84
	LTS0255728	-6.60	4.84
	LTS0031098	-6.60	4.84
	LTS0068303	-6.60	4.84
	LTS0271058	-6.60	4.84

Sources	Ligands (LOTUS ID)	Binding Free Energy (kcal/mol)	pKi
	LTS0107505	-6.60	4.84
	LTS0265960	-6.60	4.84
	LTS0031808	-6.60	4.84
	LTS0036180	-6.50	4.77
	LTS0241120	-6.50	4.77
	LTS0108018	-6.50	4.77
	LTS0184013	-6.50	4.77
	LTS0166378	-6.50	4.77
	LTS0169132	-6.40	4.69
	LTS0248258	-6.40	4.69
	LTS0168849	-6.40	4.69
	LTS0175293	-6.40	4.69
	LTS0217700	-6.40	4.69
	LTS0101481	-6.40	4.69
	LTS0105772	-6.40	4.69
	LTS0118210	-6.30	4.62
	LTS0226959	-6.30	4.62
	LTS0268790	-6.30	4.62
	LTS0075274	-6.30	4.62
	LTS0106601	-6.30	4.62
	LTS0232226	-6.30	4.62
	LTS0153976	-6.30	4.62
	LTS0070101	-6.30	4.62
	LTS0119848	-6.30	4.62
	LTS0253341	-6.30	4.62
	LTS0191258	-6.30	4.62
	LTS0062007	-6.20	4.55
	LTS0052320	-6.20	4.55
	LTS0073517	-6.10	4.47
	LTS0127622	-6.10	4.47
	LTS0127734	-6.10	4.47
	LTS0235447	-6.10	4.47

Sources	Ligands (LOTUS ID)	Binding Free Energy (kcal/mol)	pKi
	LTS0091612	-6.00	4.40
	LTS0119894	-6.00	4.40
	LTS0258092	-6.00	4.40
	LTS0155981	-5.80	4.25
	LTS0230308	-5.80	4.25
	LTS0238038	-5.80	4.25
	LTS0110409	-5.70	4.18
	LTS0214036	-5.70	4.18
	LTS0241025	-5.70	4.18
	LTS0107907	-5.60	4.11
	LTS0181568	-5.30	3.89
	LTS0124668	-5.00	3.67
	LTS0071072	-5.00	3.67
	LTS0020478	-13.40	9.83
	LTS0076848	-13.40	9.83
	LTS0164739	-13.40	9.83
	LTS0210066	-13.40	9.83
	LTS0222826	-13.40	9.83
	LTS0256994	-13.40	9.83
	LTS0024855	-12.70	9.31
	LTS0026037	-12.70	9.31
	LTS0037971	-12.70	9.31
	LTS0165521	-12.70	9.31
	LTS0186040	-12.70	9.31
	LTS0190545	-12.70	9.31
	LTS0067513	-12.60	9.24
	LTS0255462	-12.60	9.24
	LTS0058728	-12.50	9.17
	LTS0102008	-12.50	9.17
	LTS0145581	-12.50	9.17
	LTS0087735	-12.30	9.02
	LTS0049118	-12.20	8.95

Sources	Ligands (LOTUS ID)	Binding Free Energy (kcal/mol)	pKi
	LTS0252412	-12.20	8.95
	LTS0209872	-12.10	8.87
	LTS0228971	-12.10	8.87
	LTS0194342	-12.00	8.80
	LTS0204838	-12.00	8.80
	LTS0000431	-12.00	8.80
	LTS0149962	-12.00	8.80
	LTS0071482	-11.90	8.73
	LTS0055667	-11.80	8.65
	LTS0189037	-11.80	8.65
	LTS0099758	-11.80	8.65
	LTS0017129	-11.70	8.58
	LTS0136842	-11.70	8.58
	LTS0053191	-11.60	8.51
	LTS0057277	-11.60	8.51
	LTS0124152	-11.60	8.51
	LTS0147801	-11.60	8.51
	LTS0211258	-11.60	8.51
	LTS0225602	-11.60	8.51
	LTS0233386	-11.60	8.51
	LTS0245598	-11.60	8.51
	LTS0186018	-11.50	8.43
	LTS0011220	-11.30	8.29
	LTS0092918	-11.10	8.14
	LTS0078621	-11.00	8.07
	LTS0216697	-11.00	8.07
	LTS0039680	-11.00	8.07
	LTS0199299	-10.90	7.99
	LTS0204457	-10.70	7.85
	LTS0225628	-10.70	7.85
	LTS0108364	-10.70	7.85
	LTS0140569	-10.40	7.63

Sources	Ligands (LOTUS ID)	Binding Free Energy (kcal/mol)	pKi
	LTS0054435	-10.20	7.48
	LTS0177009	-9.90	7.26
	LTS0251864	-9.90	7.26
	LTS0186212	-9.70	7.11
	LTS0200986	-9.60	7.04
	LTS0084570	-9.50	6.97
	LTS0200974	-9.40	6.89
	LTS0030061	-8.90	6.53
	LTS0036534	-8.90	6.53
	LTS0051316	-8.90	6.53
	LTS0100543	-8.90	6.53
	LTS0125858	-8.90	6.53
	LTS0207603	-8.90	6.53
	LTS0225131	-8.90	6.53
	LTS0071158	-8.90	6.53
	LTS0046493	-8.80	6.45
	LTS0146961	-8.80	6.45
	LTS0013347	-8.70	6.38
	LTS0072671	-8.40	6.16
	LTS0029311	-8.10	5.94
	LTS0263446	-8.00	5.87
	LTS0262894	-7.90	5.79
	LTS0204616	-7.90	5.79
	LTS0125608	-7.70	5.65
	LTS0215205	-7.70	5.65
	LTS0210386	-7.30	5.35
	LTS0260373	-7.20	5.28
	LTS0071224	-7.10	5.21
	LTS0207208	-7.10	5.21
	LTS0225703	-5.40	3.96
	LTS0189346	-4.50	3.30
**Foeniculum vulgare**	LTS0087204	-14.20	10.41

Sources	Ligands (LOTUS ID)	Binding Free Energy (kcal/mol)	pKi
	LTS0141130	-14.20	10.41
	LTS0117717	-13.90	10.19
	LTS0036839	-11.90	8.73
	LTS0259097	-11.10	8.14
	LTS0121768	-10.90	7.99
	LTS0180235	-10.90	7.99
	LTS0226495	-10.70	7.85
	LTS0232027	-10.60	7.77
	LTS0066242	-10.40	7.63
	LTS0119144	-10.40	7.63
	LTS0211330	-10.20	7.48
	LTS0044326	-10.00	7.33
	LTS0092046	-10.00	7.33
	LTS0082206	-9.90	7.26
	LTS0233400	-9.90	7.26
	LTS0095810	-9.80	7.19
	LTS0112986	-9.60	7.04
	LTS0119798	-9.60	7.04
	LTS0056761	-9.40	6.89
	LTS0088320	-9.40	6.89
	LTS0101022	-9.40	6.89
	LTS0129721	-9.40	6.89
	LTS0157527	-9.40	6.89
	LTS0187063	-9.40	6.89
	LTS0210216	-9.40	6.89
	LTS0210363	-9.40	6.89
	LTS0240143	-9.40	6.89
	LTS0138220	-9.30	6.82
	LTS0187739	-9.30	6.82
	LTS0128144	-9.20	6.75
	LTS0212143	-9.20	6.75
	LTS0091254	-9.10	6.67

Sources	Ligands (LOTUS ID)	Binding Free Energy (kcal/mol)	pKi
	LTS0113114	-9.10	6.67
	LTS0174624	-9.10	6.67
	LTS0230001	-9.10	6.67
	LTS0024754	-9.00	6.60
	LTS0124898	-9.00	6.60
	LTS0126240	-9.00	6.60
	LTS0134856	-9.00	6.60
	LTS0271830	-9.00	6.60
	LTS0042292	-8.90	6.53
	LTS0086280	-8.90	6.53
	LTS0086979	-8.90	6.53
	LTS0184698	-8.90	6.53
	LTS0220020	-8.90	6.53
	LTS0010495	-8.80	6.45
	LTS0185636	-8.80	6.45
	LTS0260944	-8.80	6.45
	LTS0032845	-8.70	6.38
	LTS0220789	-8.70	6.38
	LTS0239198	-8.70	6.38
	LTS0007372	-8.60	6.31
	LTS0091905	-8.60	6.31
	LTS0170558	-8.60	6.31
	LTS0178847	-8.60	6.31
	LTS0090221	-8.50	6.23
	LTS0093095	-8.50	6.23
	LTS0093611	-8.50	6.23
	LTS0108757	-8.50	6.23
	LTS0186298	-8.50	6.23
	LTS0211340	-8.50	6.23
	LTS0270485	-8.50	6.23
	LTS0034490	-8.40	6.16
	LTS0045574	-8.40	6.16

Prediction of Single Nucleotide Polymorphisms of RNA Dependent RNA Polymerase for the Potato Leafroll Virus Using Computational and Experimental Approaches

Sources	Ligands (LOTUS ID)	Binding Free Energy (kcal/mol)	pKi
	LTS0088971	-8.40	6.16
	LTS0117550	-8.40	6.16
	LTS0126936	-8.40	6.16
	LTS0130521	-8.40	6.16
	LTS0132416	-8.40	6.16
	LTS0157545	-8.40	6.16
	LTS0168861	-8.40	6.16
	LTS0203179	-8.40	6.16
	LTS0211102	-8.40	6.16
	LTS0065676	-8.30	6.09
	LTS0136060	-8.30	6.09
	LTS0149709	-8.30	6.09
	LTS0191800	-8.30	6.09
	LTS0195312	-8.30	6.09
	LTS0241372	-8.30	6.09
	LTS0254337	-8.30	6.09
	LTS0261470	-8.30	6.09
	LTS0267242	-8.30	6.09
	LTS0012002	-8.20	6.01
	LTS0015984	-8.20	6.01
	LTS0112248	-8.20	6.01
	LTS0121746	-8.20	6.01
	LTS0158828	-8.20	6.01
	LTS0168570	-8.20	6.01
	LTS0200416	-8.20	6.01
	LTS0201798	-8.20	6.01
	LTS0010529	-8.10	5.94
	LTS0012830	-8.10	5.94
	LTS0162810	-8.10	5.94
	LTS0253733	-8.10	5.94
	LTS0019861	-8.00	5.87
	LTS0022511	-8.00	5.87

Sources	Ligands (LOTUS ID)	Binding Free Energy (kcal/mol)	pKi
	LTS0029311	-8.00	5.87
	LTS0087575	-8.00	5.87
	LTS0137002	-8.00	5.87
	LTS0186947	-8.00	5.87
	LTS0206339	-8.00	5.87
	LTS0224133	-8.00	5.87
	LTS0261050	-8.00	5.87
	LTS0020694	-7.90	5.79
	LTS0041825	-7.90	5.79
	LTS0086962	-7.90	5.79
	LTS0091675	-7.90	5.79
	LTS0126716	-7.90	5.79
	LTS0027487	-7.80	5.72
	LTS0074451	-7.80	5.72
	LTS0000771	-7.70	5.65
	LTS0064424	-7.70	5.65
	LTS0158691	-7.70	5.65
	LTS0193112	-7.70	5.65
	LTS0194928	-7.70	5.65
	LTS0274535	-7.70	5.65
	LTS0172442	-7.60	5.57
	LTS0220125	-7.60	5.57
	LTS0229892	-7.60	5.57
	LTS0076625	-7.50	5.50
	LTS0089480	-7.50	5.50
	LTS0241170	-7.50	5.50
	LTS0083430	-7.40	5.43
	LTS0143168	-7.40	5.43
	LTS0134620	-7.30	5.35
	LTS0151391	-7.30	5.35
	LTS0217852	-7.30	5.35
	LTS0218223	-7.30	5.35

Sources	Ligands (LOTUS ID)	Binding Free Energy (kcal/mol)	pKi
	LTS0221448	-7.30	5.35
	LTS0010038	-7.20	5.28
	LTS0031219	-7.20	5.28
	LTS0044375	-7.20	5.28
	LTS0052817	-7.20	5.28
	LTS0079421	-7.20	5.28
	LTS0079439	-7.20	5.28
	LTS0098583	-7.20	5.28
	LTS0154729	-7.20	5.28
	LTS0159739	-7.20	5.28
	LTS0163918	-7.20	5.28
	LTS0185993	-7.20	5.28
	LTS0221589	-7.20	5.28
	LTS0234203	-7.20	5.28
	LTS0242381	-7.20	5.28
	LTS0071224	-7.10	5.21
	LTS0106847	-7.10	5.21
	LTS0145723	-7.10	5.21
	LTS0204616	-7.10	5.21
	LTS0258838	-7.10	5.21
	LTS0028421	-7.00	5.13
	LTS0035117	-7.00	5.13
	LTS0153666	-7.00	5.13
	LTS0176031	-7.00	5.13
	LTS0015558	-6.90	5.06
	LTS0067708	-6.90	5.06
	LTS0115731	-6.90	5.06
	LTS0137694	-6.80	4.99
	LTS0210495	-6.80	4.99
	LTS0017052	-6.70	4.91
	LTS0065297	-6.70	4.91
	LTS0085212	-6.70	4.91

Sources	Ligands (LOTUS ID)	Binding Free Energy (kcal/mol)	pKi
	LTS0120009	-6.70	4.91
	LTS0139858	-6.70	4.91
	LTS0263171	-6.70	4.91
	LTS0092442	-6.60	4.84
	LTS0104946	-6.60	4.84
	LTS0107456	-6.60	4.84
	LTS0107505	-6.60	4.84
	LTS0155822	-6.60	4.84
	LTS0172890	-6.60	4.84
	LTS0245226	-6.60	4.84
	LTS0004651	-6.50	4.77
	LTS0088932	-6.40	4.69
	LTS0174687	-6.40	4.69
	LTS0027481	-6.30	4.62
	LTS0033696	-6.30	4.62
	LTS0090925	-6.30	4.62
	LTS0119764	-6.30	4.62
	LTS0128050	-6.30	4.62
	LTS0182827	-6.30	4.62
	LTS0042890	-6.20	4.55
	LTS0142944	-6.20	4.55
	LTS0164574	-6.20	4.55
	LTS0023739	-6.10	4.47
	LTS0049458	-6.10	4.47
	LTS0077105	-6.10	4.47
	LTS0134742	-6.10	4.47
	LTS0272557	-6.10	4.47
	LTS0009997	-6.00	4.40
	LTS0097726	-6.00	4.40
	LTS0123492	-6.00	4.40
	LTS0140500	-6.00	4.40
	LTS0223315	-6.00	4.40

Sources	Ligands (LOTUS ID)	Binding Free Energy (kcal/mol)	pKi
	LTS0248038	-6.00	4.40
	LTS0101823	-5.90	4.33
	LTS0268947	-5.90	4.33
	LTS0057266	-5.80	4.25
	LTS0085864	-5.80	4.25
	LTS0155981	-5.80	4.25
	LTS0167727	-5.80	4.25
	LTS0177188	-5.80	4.25
	LTS0199582	-5.80	4.25
	LTS0202484	-5.80	4.25
	LTS0205325	-5.80	4.25
	LTS0208391	-5.80	4.25
	LTS0244943	-5.80	4.25
	LTS0151982	-5.70	4.18
	LTS0156471	-5.70	4.18
	LTS0188912	-5.70	4.18
	LTS0190477	-5.70	4.18
	LTS0196605	-5.70	4.18
	LTS0197428	-5.70	4.18
	LTS0208598	-5.70	4.18
	LTS0242576	-5.70	4.18
	LTS0046227	-5.60	4.11
	LTS0259277	-5.60	4.11
	LTS0275578	-5.60	4.11
	LTS0117046	-5.50	4.03
	LTS0175104	-5.50	4.03
	LTS0185307	-5.50	4.03
	LTS0062819	-5.40	3.96
	LTS0090341	-5.40	3.96
	LTS0113372	-5.40	3.96
	LTS0114247	-5.40	3.96
	LTS0232891	-5.40	3.96

Sources	Ligands (LOTUS ID)	Binding Free Energy (kcal/mol)	pKi
	LTS0241114	-5.40	3.96
	LTS0273091	-5.40	3.96
	LTS0027671	-5.30	3.89
	LTS0054560	-5.30	3.89
	LTS0058950	-5.30	3.89
	LTS0060376	-5.30	3.89
	LTS0086394	-5.30	3.89
	LTS0090353	-5.30	3.89
	LTS0175810	-5.30	3.89
	LTS0199986	-5.30	3.89
	LTS0207920	-5.30	3.89
	LTS0263183	-5.30	3.89
	LTS0014061	-5.20	3.81
	LTS0121039	-5.20	3.81
	LTS0226629	-5.20	3.81
	LTS0262158	-5.20	3.81
	LTS0045973	-5.10	3.74
	LTS0076097	-5.10	3.74
	LTS0161796	-5.10	3.74
	LTS0226542	-5.10	3.74
	LTS0242970	-5.10	3.74
	LTS0259218	-5.10	3.74
	LTS0274578	-5.10	3.74
	LTS0013597	-5.00	3.67
	LTS0124668	-5.00	3.67
	LTS0157173	-5.00	3.67
	LTS0174739	-5.00	3.67
	LTS0178482	-5.00	3.67
	LTS0181568	-5.00	3.67
	LTS0184149	-5.00	3.67
	LTS0185507	-5.00	3.67
	LTS0200037	-5.00	3.67

Sources	Ligands (LOTUS ID)	Binding Free Energy (kcal/mol)	pKi
	LTS0076732	-4.90	3.59
	LTS0104525	-4.90	3.59
	LTS0151306	-4.90	3.59
	LTS0170891	-4.90	3.59
	LTS0255838	-4.90	3.59
	LTS0008205	-4.80	3.52
	LTS0147438	-4.80	3.52
	LTS0153918	-4.80	3.52
	LTS0161675	-4.80	3.52
	LTS0203323	-4.80	3.52
	LTS0013198	-4.70	3.45
	LTS0229113	-4.70	3.45
	LTS0230612	-4.70	3.45
	LTS0040648	-4.60	3.37
	LTS0190325	-4.60	3.37
	LTS0213858	-4.60	3.37
	LTS0088233	-4.50	3.30
	LTS0256910	-4.50	3.30
	LTS0028766	-4.40	3.23
	LTS0112297	-4.40	3.23
	LTS0141759	-4.40	3.23
	LTS0151848	-4.40	3.23
	LTS0257971	-4.40	3.23
	LTS0013195	-4.30	3.15
	LTS0105148	-4.10	3.01
	LTS0146158	-4.10	3.01
	LTS0150163	-4.10	3.01
	LTS0250310	-3.90	2.86
	LTS0155285	-3.80	2.79
	LTS0170307	-3.70	2.71
	LTS0054613	-3.40	2.49
	LTS0123435	-14.80	10.86

Sources	Ligands (LOTUS ID)	Binding Free Energy (kcal/mol)	pKi
**Syzygium aromaticum**	LTS0096540	-14.50	10.64
	LTS0241644	-14.50	10.64
**Syzygium aromaticum**	LTS0009009	-14.50	10.64
**Syzygium aromaticum**	LTS0087204	-14.30	10.49
	LTS0105563	-13.30	9.75
**Syzygium aromaticum**	LTS0216151	-13.30	9.75
**Syzygium aromaticum**	LTS0122912	-13.20	9.68
	LTS0090883	-13.10	9.61
**Syzygium aromaticum**	LTS0041901	-13.10	9.61
**Syzygium aromaticum**	LTS0109701	-12.90	9.46
	LTS0117717	-12.90	9.46
**Syzygium aromaticum**	LTS0090227	-12.00	8.80
	LTS0048713	-12.00	8.80
**Syzygium aromaticum**	LTS0269672	-11.70	8.58
**Syzygium aromaticum**	LTS0233186	-11.40	8.36
	LTS0016187	-11.40	8.36
**Syzygium aromaticum**	LTS0117174	-11.30	8.29
**Syzygium aromaticum**	LTS0097375	-11.20	8.21
	LTS0207554	-10.90	7.99
**Syzygium aromaticum**	LTS0247872	-10.80	7.92
**Syzygium aromaticum**	LTS0137113	-10.70	7.85
	LTS0137319	-10.70	7.85
**Syzygium aromaticum**	LTS0142264	-10.70	7.85
**Syzygium aromaticum**	LTS0156299	-10.70	7.85
	LTS0153830	-10.70	7.85
**Syzygium aromaticum**	LTS0048403	-10.70	7.85
	LTS0064840	-10.70	7.85
**Syzygium aromaticum**	LTS0159314	-10.60	7.77
**Syzygium aromaticum**	LTS0229223	-10.60	7.77
	LTS0207598	-10.50	7.70
**Syzygium aromaticum**	LTS0266849	-10.50	7.70
	LTS0007322	-10.50	7.70

Sources	Ligands (LOTUS ID)	Binding Free Energy (kcal/mol)	pKi
**Syzygium aromaticum**	LTS0067445	-10.50	7.70
	LTS0190031	-10.30	7.55
**Syzygium aromaticum**	LTS0091334	-10.30	7.55
**Syzygium aromaticum**	LTS0149780	-10.30	7.55
	LTS0073168	-10.30	7.55
**Syzygium aromaticum**	LTS0207820	-10.20	7.48
**Syzygium aromaticum**	LTS0090331	-10.00	7.33
	LTS0259697	-10.00	7.33
**Syzygium aromaticum**	LTS0198024	-9.60	7.04
**Syzygium aromaticum**	LTS0096341	-9.50	6.97
	LTS0064715	-9.40	6.89
**Syzygium aromaticum**	LTS0139634	-9.10	6.67
	LTS0228241	-9.10	6.67
**Syzygium aromaticum**	LTS0052920	-9.10	6.67
**Syzygium aromaticum**	LTS0232017	-9.00	6.60
	LTS0126471	-9.00	6.60
**Syzygium aromaticum**	LTS0028499	-9.00	6.60
**Syzygium aromaticum**	LTS0114316	-8.90	6.53
	LTS0234952	-8.90	6.53
**Syzygium aromaticum**	LTS0053115	-8.90	6.53
**Syzygium aromaticum**	LTS0103949	-8.80	6.45
	LTS0111070	-8.80	6.45
**Syzygium aromaticum**	LTS0154650	-8.80	6.45
**Syzygium aromaticum**	LTS0270743	-8.80	6.45
	LTS0088971	-8.80	6.45
**Syzygium aromaticum**	LTS0130248	-8.80	6.45
	LTS0019321	-8.80	6.45
**Syzygium aromaticum**	LTS0022607	-8.80	6.45
**Syzygium aromaticum**	LTS0023424	-8.80	6.45
	LTS0216134	-8.70	6.38
**Syzygium aromaticum**	LTS0226232	-8.70	6.38
	LTS0117550	-8.40	6.16

Sources	Ligands (LOTUS ID)	Binding Free Energy (kcal/mol)	pKi
**Syzygium aromaticum**	LTS0250499	-8.40	6.16
**Syzygium aromaticum**	LTS0160659	-8.40	6.16
	LTS0007306	-8.40	6.16
**Syzygium aromaticum**	LTS0022493	-8.40	6.16
**Syzygium aromaticum**	LTS0126716	-8.30	6.09
	LTS0241346	-8.30	6.09
**Syzygium aromaticum**	LTS0084305	-8.20	6.01
	LTS0076809	-8.20	6.01
**Syzygium aromaticum**	LTS0186293	-8.20	6.01
**Syzygium aromaticum**	LTS0056682	-8.20	6.01
	LTS0253916	-8.10	5.94
**Syzygium aromaticum**	LTS0080319	-8.10	5.94
**Syzygium aromaticum**	LTS0152670	-8.10	5.94
	LTS0095103	-8.10	5.94
**Syzygium aromaticum**	LTS0025893	-8.10	5.94
	LTS0030886	-8.10	5.94
**Syzygium aromaticum**	LTS0185078	-8.00	5.87
**Syzygium aromaticum**	LTS0219322	-8.00	5.87
	LTS0249995	-7.90	5.79
**Syzygium aromaticum**	LTS0260880	-7.90	5.79
**Syzygium aromaticum**	LTS0125218	-7.90	5.79
	LTS0116588	-7.80	5.72
**Syzygium aromaticum**	LTS0222425	-7.70	5.65
**Syzygium aromaticum**	LTS0055913	-7.70	5.65
	LTS0176954	-7.60	5.57
**Syzygium aromaticum**	LTS0028605	-7.60	5.57
**Syzygium aromaticum**	LTS0255026	-7.50	5.50
	LTS0207256	-7.50	5.50
**Syzygium aromaticum**	LTS0221686	-7.50	5.50
	LTS0029429	-7.30	5.35
**Syzygium aromaticum**	LTS0071224	-7.30	5.35
	LTS0126973	-7.20	5.28

Sources	Ligands (LOTUS ID)	Binding Free Energy (kcal/mol)	pKi
	LTS0049765	-7.20	5.28
	LTS0098771	-7.10	5.21
	LTS0204616	-7.10	5.21
	LTS0168527	-7.00	5.13
	LTS0246122	-7.00	5.13
	LTS0140691	-6.90	5.06
	LTS0141423	-6.90	5.06
	LTS0263005	-6.90	5.06
	LTS0092876	-6.90	5.06
	LTS0131870	-6.80	4.99
	LTS0086128	-6.80	4.99
	LTS0136836	-6.80	4.99
	LTS0183510	-6.80	4.99
	LTS0004651	-6.80	4.99
	LTS0009773	-6.80	4.99
	LTS0085212	-6.70	4.91
	LTS0263171	-6.70	4.91
	LTS0139858	-6.70	4.91
	LTS0117079	-6.60	4.84
	LTS0155822	-6.60	4.84
	LTS0116177	-6.60	4.84
	LTS0042045	-6.60	4.84
	LTS0008864	-6.60	4.84
	LTS0159789	-6.40	4.69
	LTS0213960	-6.40	4.69
	LTS0097515	-6.40	4.69
	LTS0136437	-6.30	4.62
	LTS0128050	-6.30	4.62
	LTS0255317	-6.30	4.62
	LTS0125222	-6.20	4.55
	LTS0148717	-6.20	4.55
	LTS0171573	-6.20	4.55

Sources	Ligands (LOTUS ID)	Binding Free Energy (kcal/mol)	pKi
	LTS0037297	-6.20	4.55
	LTS0008282	-6.20	4.55
	LTS0124278	-6.10	4.47
	LTS0233023	-6.10	4.47
	LTS0266252	-6.10	4.47
	LTS0211245	-6.10	4.47
	LTS0241073	-6.10	4.47
	LTS0248056	-6.00	4.40
	LTS0254484	-5.90	4.33
	LTS0104731	-5.90	4.33
	LTS0225398	-5.90	4.33
	LTS0245014	-5.90	4.33
	LTS0116548	-5.80	4.25
	LTS0155971	-5.80	4.25
	LTS0098881	-5.80	4.25
	LTS0244260	-5.70	4.18
	LTS0129079	-5.60	4.11
	LTS0018765	-5.60	4.11
	LTS0033696	-5.60	4.11
	LTS0177188	-5.40	3.96
	LTS0037806	-5.40	3.96
	LTS0222857	-5.30	3.89
	LTS0245226	-5.30	3.89
	LTS0087207	-5.30	3.89
	LTS0012882	-5.30	3.89
	LTS0186625	-5.20	3.81
	LTS0217393	-5.20	3.81
	LTS0228439	-5.20	3.81
	LTS0094193	-5.10	3.74
	LTS0165886	-5.10	3.74
	LTS0139222	-5.10	3.74
	LTS0038574	-5.10	3.74

Sources	Ligands (LOTUS ID)	Binding Free Energy (kcal/mol)	pKi
	LTS0106377	-5.00	3.67
	LTS0108749	-5.00	3.67
	LTS0204713	-4.90	3.59
	LTS0210036	-4.80	3.52
	LTS0052342	-4.80	3.52
	LTS0238624	-4.70	3.45
	LTS0049015	-4.60	3.37
	LTS0143969	-4.50	3.30
	LTS0165799	-4.20	3.08
**Thuja orientalis**	LTS0126730	-14.10	10.34
	LTS0009479	-13.50	9.90
	LTS0000106	-12.80	9.39
	LTS0007966	-12.80	9.39
	LTS0014004	-12.50	9.17
	LTS0025882	-12.40	9.09
	LTS0183855	-12.10	8.87
	LTS0110045	-11.80	8.65
	LTS0213238	-11.20	8.21
	LTS0187644	-11.10	8.14
	LTS0198297	-10.90	7.99
	LTS0238294	-10.80	7.92
	LTS0063796	-10.80	7.92
	LTS0082087	-10.80	7.92
	LTS0193829	-10.80	7.92
	LTS0206321	-10.70	7.85
	LTS0206999	-10.60	7.77
	LTS0254320	-10.40	7.63
	LTS0138463	-10.30	7.55
	LTS0086765	-10.30	7.55
	LTS0009856	-10.20	7.48
	LTS0162062	-10.20	7.48
	LTS0184527	-10.10	7.41

Sources	Ligands (LOTUS ID)	Binding Free Energy (kcal/mol)	pKi
	LTS0221015	-10.10	7.41
	LTS0046281	-10.00	7.33
	LTS0251071	-10.00	7.33
	LTS0146988	-10.00	7.33
	LTS0072238	-10.00	7.33
	LTS0108903	-10.00	7.33
	LTS0009064	-9.90	7.26
	LTS0153299	-9.90	7.26
	LTS0030228	-9.80	7.19
	LTS0161139	-9.80	7.19
	LTS0208774	-9.80	7.19
	LTS0134832	-9.70	7.11
	LTS0262609	-9.70	7.11
	LTS0193604	-9.70	7.11
	LTS0246825	-9.50	6.97
	LTS0092282	-9.30	6.82
	LTS0159867	-9.30	6.82
	LTS0065195	-9.30	6.82
	LTS0050471	-9.10	6.67
	LTS0083162	-9.10	6.67
	LTS0160820	-9.10	6.67
	LTS0069360	-8.90	6.53
	LTS0087335	-8.90	6.53
	LTS0146881	-8.90	6.53
	LTS0057912	-8.90	6.53
	LTS0100511	-8.80	6.45
	LTS0137465	-8.80	6.45
	LTS0274482	-8.70	6.38
	LTS0228629	-8.60	6.31
	LTS0088971	-8.60	6.31
	LTS0015240	-8.50	6.23
	LTS0132416	-8.50	6.23

Sources	Ligands (LOTUS ID)	Binding Free Energy (kcal/mol)	pKi
	LTS0132985	-8.50	6.23
	LTS0171587	-8.50	6.23
	LTS0062135	-8.50	6.23
	LTS0093095	-8.50	6.23
	LTS0107899	-8.50	6.23
	LTS0137007	-8.50	6.23
	LTS0223445	-8.50	6.23
	LTS0158828	-8.40	6.16
	LTS0186298	-8.30	6.09
	LTS0025540	-8.30	6.09
	LTS0189989	-8.20	6.01
	LTS0253733	-8.10	5.94
	LTS0176954	-8.00	5.87
	LTS0224133	-8.00	5.87
	LTS0013249	-8.00	5.87
	LTS0143348	-8.00	5.87
	LTS0255618	-8.00	5.87
	LTS0024262	-8.00	5.87
	LTS0029311	-8.00	5.87
	LTS0201798	-7.80	5.72
	LTS0085212	-7.70	5.65
	LTS0071224	-7.70	5.65
	LTS0204616	-7.70	5.65
	LTS0114195	-7.60	5.57
	LTS0173497	-7.60	5.57
	LTS0070589	-7.50	5.50
	LTS0237766	-7.50	5.50
	LTS0139658	-7.50	5.50
	LTS0019525	-7.40	5.43
	LTS0209662	-7.40	5.43
	LTS0217146	-7.40	5.43
	LTS0250522	-7.40	5.43

Sources	Ligands (LOTUS ID)	Binding Free Energy (kcal/mol)	pKi
	LTS0251466	-7.40	5.43
	LTS0063367	-7.40	5.43
	LTS0168085	-7.40	5.43
	LTS0207129	-7.40	5.43
	LTS0247226	-7.40	5.43
	LTS0266901	-7.40	5.43
	LTS0079439	-7.40	5.43
	LTS0045608	-7.30	5.35
	LTS0067851	-7.30	5.35
	LTS0092659	-7.30	5.35
	LTS0236863	-7.30	5.35
	LTS0251712	-7.30	5.35
	LTS0101019	-7.30	5.35
	LTS0121523	-7.30	5.35
	LTS0141224	-7.30	5.35
	LTS0158625	-7.30	5.35
	LTS0258280	-7.30	5.35
	LTS0166814	-7.30	5.35
	LTS0273927	-7.20	5.28
	LTS0135073	-7.20	5.28
	LTS0021824	-7.10	5.21
	LTS0202406	-7.10	5.21
	LTS0262016	-7.10	5.21
	LTS0010485	-7.10	5.21
	LTS0017816	-7.10	5.21
	LTS0049492	-7.10	5.21
	LTS0168132	-7.10	5.21
	LTS0239717	-7.10	5.21
	LTS0097200	-7.00	5.13
	LTS0175490	-7.00	5.13
	LTS0201900	-7.00	5.13
	LTS0267961	-7.00	5.13

Sources	Ligands (LOTUS ID)	Binding Free Energy (kcal/mol)	pKi
	LTS0268318	-7.00	5.13
	LTS0077328	-7.00	5.13
	LTS0079794	-7.00	5.13
	LTS0097230	-7.00	5.13
	LTS0164806	-7.00	5.13
	LTS0181981	-6.90	5.06
	LTS0184139	-6.90	5.06
	LTS0041563	-6.90	5.06
	LTS0057750	-6.90	5.06
	LTS0115731	-6.90	5.06
	LTS0155854	-6.90	5.06
	LTS0109693	-6.90	5.06
	LTS0017052	-6.80	4.99
	LTS0027694	-6.80	4.99
	LTS0263171	-6.70	4.91
	LTS0004651	-6.70	4.91
	LTS0028747	-6.70	4.91
	LTS0007173	-6.70	4.91
	LTS0104946	-6.60	4.84
	LTS0139858	-6.60	4.84
	LTS0182752	-6.60	4.84
	LTS0196591	-6.60	4.84
	LTS0222761	-6.60	4.84
	LTS0004456	-6.50	4.77
	LTS0001843	-6.50	4.77
	LTS0210862	-6.50	4.77
	LTS0106819	-6.50	4.77
	LTS0172943	-6.40	4.69
	LTS0247962	-6.40	4.69
	LTS0221297	-6.20	4.55
	LTS0155981	-5.80	4.25
	LTS0112569	-5.70	4.18

Sources	Ligands (LOTUS ID)	Binding Free Energy (kcal/mol)	pKi
	LTS0106881	-5.60	4.11
	LTS0217821	-5.60	4.11
	LTS0104525	-5.20	3.81
	LTS0139222	-5.20	3.81
	LTS0266252	-5.10	3.74
	LTS0073904	-5.00	3.67
	LTS0157173	-4.90	3.59
	LTS0234389	-4.80	3.52
	LTS0028668	-4.70	3.45
	LTS0171944	-4.60	3.37
	LTS0065505	-4.50	3.30
	LTS0136368	-4.40	3.23
	LTS0194724	-4.30	3.15
	LTS0130979	-4.30	3.15
	LTS0245493	-4.30	3.15
	LTS0163741	-4.20	3.08
	LTS0207008	-4.20	3.08
	LTS0101459	-4.20	3.08
	LTS0140051	-4.10	3.01
	LTS0049071	-4.00	2.93
	LTS0157578	-3.80	2.79

Supplementary Table 1. Molecular docking interactions of the bioactive compounds of *Aloe vera, Artemisia campestris, Calotropis procera, Foeniculum vulgare, Syzygium aromaticum, and Thuja orientalis* against the wild-type and mutant proteins.
